# A Common Polymorphism within the IGF2 Imprinting Control Region Is Associated with Parent of Origin Specific Effects in Infantile Hemangiomas

**DOI:** 10.1371/journal.pone.0113168

**Published:** 2015-10-23

**Authors:** Brent Schultz, Xiaopan Yao, Yanhong Deng, Milton Waner, Christopher Spock, Laura Tom, John Persing, Deepak Narayan

**Affiliations:** 1 University of Washington, Division of Plastic Surgery, Seattle, WA, United States of America; 2 Yale Center for Analytic Sciences at YSPH, New Haven, CT, United States of America; 3 Vascular Birthmark Institute, New York, NY, United States of America; 4 University of Miami Hospital, Miami, FL, United States of America; 5 Division of Plastic Surgery, University of Washington, Seattle, WA, United States of America; 6 Yale Plastic and Reconstructive Surgery, New Haven, United States of America; National Cancer Institute, UNITED STATES

## Abstract

Infantile hemangioma (IH) is the most common tumor of the pediatric age group, affecting up to 4% of newborns ranging from inconsequential blemishes, to highly aggressive tumors. Following well defined growth phases (proliferative, plateau involutional) IH usually regress into a fibro-fatty residuum. Despite the high prevalence of IH, little is known regarding the pathogenesis of disease. A reported six fold decrease in IGF2 expression (correlating with transformation of proliferative to involuted lesions) prompted us to study the IGF-2 axis further. We demonstrate that IGF2 expression in IH is strongly related to the expression of a cancer testes and suspected oncogene BORIS (paralog of CTCF), placing IH in the unique category of being the first known benign BORIS positive tumor. IGF2 expression was strongly and positively related to BORIS transcript expression. Furthermore, a stronger association was made when comparing BORIS levels against the expression of CTCF via either a percentage or difference between the two. A common C/T polymorphism at CTCF BS6 appeared to modify the correlation between CTCF/BORIS and IGF2 expression in a parent of origin specific manner. Moreover, these effects may have phenotypic consequences as tumor growth also correlates with the genotype at CTCF BS6. This may provide a framework for explaining the clinical variability seen in IH and suggests new insights regarding CTCF and BORIS related functionality in both normal and malignant states.

## Introduction

Infantile hemangioma (IH) is the most common tumor of the pediatric age group, affecting up to 4% of newborns with nearly 60% localized to the head and neck[1,2]. These vascular lesions range from inconsequential blemishes to highly aggressive tumors that can threaten airways and sensorineural structures[1–6]. During the first year, hemangiomas demonstrate both histology and behavior that are also commonly noted in malignancy: immature vascular channels, high mitotic indices, and strong positivity for proliferative markers such as Ki-67 [1,5,6]. Despite these ominous beginnings, IH remain benign [1,2] The growth velocity slowly reverses leading to a “quiescent or plateau” phase of non growth (1–2 years) and then transitions into a regressive or “involuting” phase by replacing the once proliferative endothelium with a fibro-fatty residuum (2–10 years). However, these growth phases are a matter of clinical judgment alone and the exact timing of each varies considerably among studies [7]

Despite its prevalence, little is known regarding the pathogenesis of disease. Insulin Like Growth Factor 2 (IGF2) has been implicated as an important player in driving the growth of these lesions. IGF2 decreases over six fold from proliferative to involuting IH samples [8,9]. Furthermore, Beckwith-Wiedmann Syndrome (BWS), where hemangiomas are considered a supportive finding of the diagnosis—is associated with duplications or a loss of imprinting of the IGF2/H19 locus that leads to IGF2 overproduction [10,11]. Moreover, explant hemangioma cultures grow strongly in response to exogenous IGF2 [8].

IGF2 is an imprinted gene that is usually only expressed from the paternal copy [12,13]. Commonly, DNA methylation of cytosines preceding guanines (CpG’s) reinforce DNA imprinting. These so called epigenetic marks in part determine and are determined by the array of DNA binding proteins capable of interacting with specific chromatin structures. The end result of this process is diploid DNA that is potentially identical in sequence but chemically, transcriptionally and architecturally distinct in a parent of origin specific manner. This leads to activation of one given parental allele and reciprocal silencing of another [14,15]. The IGF2/H19 region of chromosome 11p15.5 serves as a model for the production of multiple imprinted transcripts[16].

Two previous studies have investigated the differential regulation and potential role of IGF2 as it pertains to IH [8,9] We carried this work further by investigating the expression of CTCF, a known chromatin insulator element for IGF2 [17,18] and it’s antagonist, BORIS (also known as CTCFL–for CTCF like) at both the transcript and protein levels. The nearby imprinted and maternally expressed H19 gene, which shares enhancers with IGF2, was also quantified. These results were then correlated with methylation analysis of key regulatory regions in the IGF2 and H19 locus. This analysis suggests that a common polymorphism within CTCF Binding Site Six, the critical imprinting control region of H19/IGF2, may have both cellular and phenotypic consequences in a parent of origin specific manner. These findings may serve as a predictor of clinical behavior of IH.

## Materials and Methods

### Ethics Statement

This study involved analyzing post excisional tissue from surgical candidates. The decision to operate was in no way influenced by this study. Clinical data was gathered retrospectively from this same group. All surgical candidates had clinical measurements available for analysis. All samples and clinical data were collected in accordance with the approved HIC protocol (#0507000430) as reviewed by the Yale University Medical School IRB. This protocol was approved specifically for this study. Written consent was obtained from each patient’s legal guardian prior to surgery. All data obtained including clinical measurements were stored in a de-identified format.

### Specimen Collection

**Fig 1:**
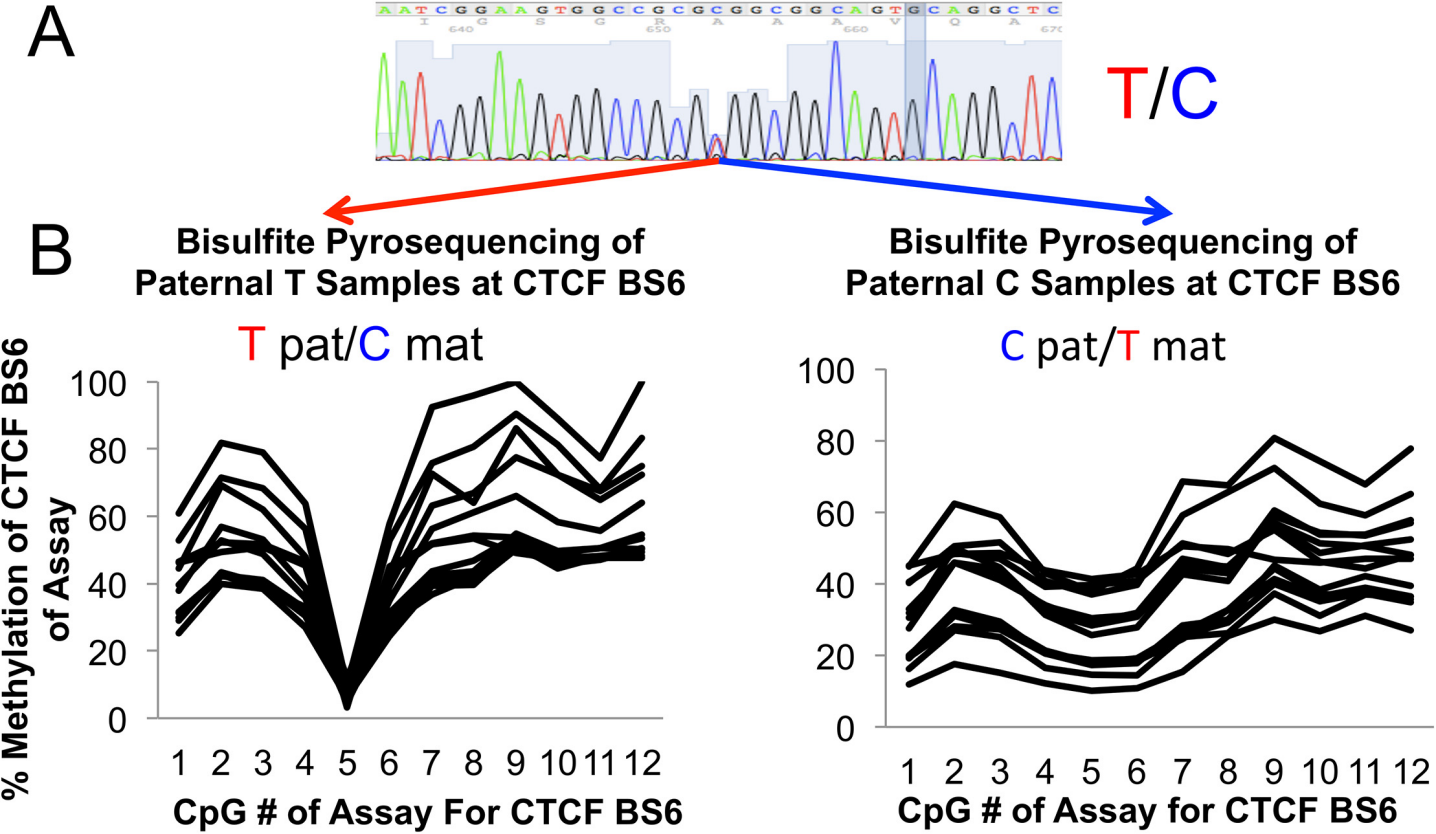
Master Data Table. All samples are assigned arbitrary numbers for ease of reference. Samples are categorized according to which set of experiments were performed, then by paternal/maternal genotype regarding the IGF2 rtPCR experiment. All sub categories are then sorted by age at resection. All quantitative data is collated with clinical descriptors. Please see methods section under specimen collection for details regarding the selection of individual samples for each experiment.

Please refer to the Master Data Table for details. Those specimens later confirmed to be hemangioma tissue, via Glut-1 positive histology, were considered for this project. Further, only discrete solitary lesions that were not found in the setting of a syndrome were considered. Those patients where prior surgical resections of the lesion were performed were also deemed ineligible. Of note, those lesions previously treated with laser were not excluded, as the effects of laser treatment are relatively superficial. However, during specimen collection, all areas that appeared grossly to be affected by laser treatment were excised before further processing. Briefly, forty-two samples were collected (See Fig 1 for details.) Of these, two samples (#41 and #42), were excluded from all analyses; sample #41 was Glut-1 negative on histology and #42 was subject to prior resections. Nineteen IH samples were selected at random for methylation analysis of the H19 promoter by southern. These 19 samples were also analyzed for methylation specific pyrosequencing of the same region. An additional two samples (numbers 1 and 14) were also subjected to H19 methylation specific pyrosequencing to bolster the number of samples with both H19 methylation and transcriptional data. Regarding transcriptional analysis, nineteen samples were found to have suitably intact RNA for quantitative RT PCR. Specimens for transcriptional analysis were separated into three categories: 1) Proliferative, 2) Quiescent, and 3) Involuting phases. As a lesion's stage is, by definition, clinical, an experienced physician staged the IH at the time of surgery. Data regarding clinical stage was gathered prospectively. The determination of the clinical stage was made by one of three highly experienced surgeons regarding vascular anomalies, using interval growth, patient age and the color/turgor of the lesion at the time of resection as criteria. General characteristics of these categories are as follows: 1) proliferative hemangiomas were generally less than 1.5 years of age with interval growth between the last two clinical visits preceding surgery, no lightening of lesional color was noted. 2) Quiescent hemangiomas: no interval growth between the last two clinic visits preceding surgery, lightening of color also played a factor in these determinations. Involuting hemangiomas: interval regression by measurement between the last two clinic visits preceding surgery, further color changes were often but not always noted.

### Clinical Data Collection

**Fig 2:**
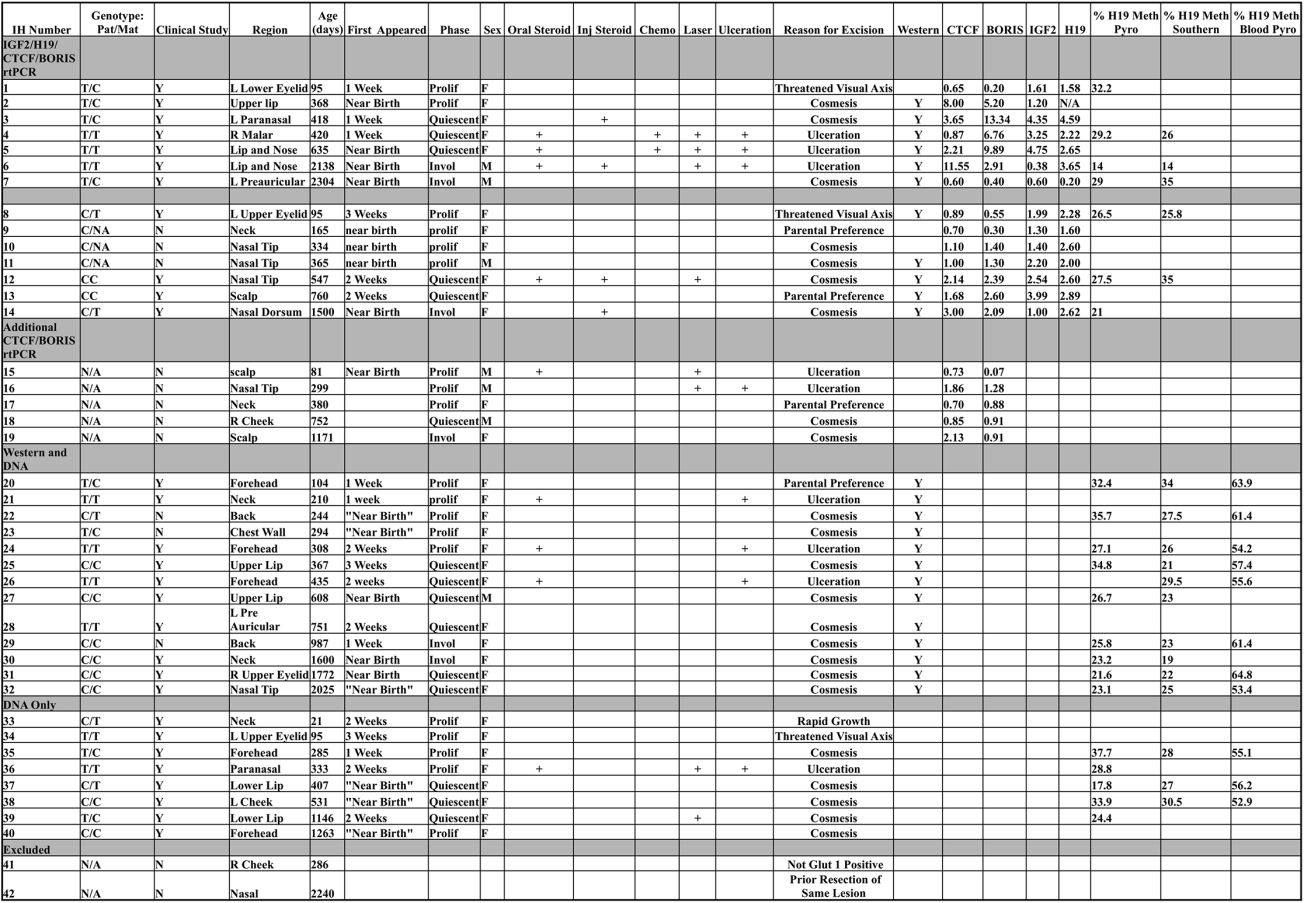
Deducing Parental Contributions From Direct Sequencing and Bisulfite Pyrosequencing. **Fig 2A:** 29 patients were genotyped via direct sequencing of blood samples for a known polymorphism within the core CTCF BS6 sequence (rs10732516.) All homozygous genotypes could be deduced from this information alone. **Fig 2B:** All samples (heterozygotes and homozygotes) were subjected to bisulfite conversion and quantitative methylation sensitive pyrosequencing. Methylation occurs only on the paternal chromosome for CTCF BS6. In normal tissue, such as patient matched control blood, this assay is capable of isolating the genotype of the paternal chromosome. As thymidine cannot be methylated, those individuals with a paternal T at rs10732516 were not methylated at CpG#5. Paternal C carrying individuals were methylated at CpG#5. Thus, the maternal and paternal contribution to CTCFBS6 can be deduced. This assay sidesteps the need for directly sequencing parents’ DNA and eliminates the potential ambiguity ensuing from hetrozygous parents. Note: The methylation values of this assay are subject to primer bias, Tost *et al (25*.*)* This is evident by the 3 distinct groupings of methylation levels, which are artifactual.

In total 34 samples were genotyped for a polymorphism within CTCF BS6 and parental contributions were determined for heterozygotes (see Fig 2 and bisulfite sequencing methods for details.) Only lesions of the head and neck were included. Of these samples, 3 were excluded because they were not on the head or neck. Two other samples were excluded because one was not Glut-1 positive on histology and one had a previous resection of the same lesion prior to evaluation, (See Fig 1 for details.) Thus 29 individuals were included in this analysis. Charts were retrospectively reviewed from patients treated either at the Yale University Plastic Surgery Center (New Haven CT), or the Vascular Birthmarks Institute (New York, New York). The age of the lesion was then compared to the size of the lesion as determined below. These data were plotted and separated by CTCF BS6 genotype and parental contribution in heterozygotes. ANCOVA analyses were then performed on putative growth curves. The age at the time of resection, with corresponding size, was used only for proliferating lesions. For those lesions that were resected at the time of involution, or quiescence, the size of the lesion at the clinical visit where quiescence was first noted was used. In the case of medical interventions, the size of the lesion before a response was noted was used. This information was used to create a clinical table of results were factors such as ulceration, steroid/chemotherapeutic, and laser treatment were also noted (note that no beta blockers were used in the sample population.) Thus, different ages are associated with most individuals when comparing the clinical data table and the master data table. For instance: Sample #7 has an age of 2304 days assigned in the Master Data Table. However, in the Clinical Table the age assigned to sample #7 is 1050 days. The difference in age assignments represent the age of the patient when the lesion was excised (this age was used for the molecular analyses) versus the age of the patient either before the first response to medical intervention was noted or when the lesion first entered the quiescent phase. Thus, for sample number 7: The lesion entered the quiescent phase (as determined retrospectively) at age 1050 days but was then excised at age 2304 days. Regarding the assessment of lesion size, if multiple dimensions were given, the largest was used. In some cases, only one dimension was given so volumetric estimations could not be calculated for every patient. Thus, patients’ lesions were standardized to a greatest diameter equivalent. This measurement was correlated with clinical photographs when available. All data utilized varied by less than 10% between stated measurement and photographic estimation when available. Lesions were classified into one of three growth phases: proliferative, quiescent and involuting. Only sizes of lesions that were in the proliferative or quiescent phase were used in this study. All data was stored in a de-identified format with a unique accession number for each patient.

### DNA Preservation and Extraction

Immediately following tissue resection, DNA was isolated using the Qiagen DNeasy Tissue Mini Kit according to the manufacturer’s protocol. Only samples with an A260/A280 measurement of 1.8 or above that ran as a single band on the gel were further analyzed.

### RNA Preservation and Extraction

Immediately following tissue resection, 100-500mg of tissue was stored in Quiagen RNA Later solution according to the manufacturer’s protocol. RNA was extracted via liquid nitrogen powder homogenization using Invitrogen Trizol reagent according to the standard protocol. 10 μg of total RNA from each sample was then treated with DNase Qiagen mini-elute columns according to manufacturer's specifications. RNA integrity was then assessed using Agilent bioanalyzer 2100 (provided as a service of the Keck Center at Yale University.) Those samples with 18s/28s ratio of 1.8 or greater were converted into cDNA using the ABI 4368813 cDNA archive kit. All samples were then stored at -80 degrees C.

### Quantitative rtPCR for CTCF, BORIS, H19 and IGF2

19 IH samples with suitable RNA, as previously specified, were subjected to fluorescent quantitative RT-PCR using ABI Taqman primers that were previously validated by the manufacturer and spanned intron exon boundaries. For reasons of sample scarcity, not all samples were subjected to each assay (See Fig 1 for details.) The assays were: IGF2—assay number Hs00171254_m1, H19—assay number Hs00399294_g1, CTCF—assay number Hs00198081_m1, and BORIS—assay number Hs00540744_m1. Gene quantification was performed using the standard curve method via pooled sample cDNA (equal contributions from each sample) and successive two fold dilutions, beginning from 50 ng and ending with 0.39 ng. All reactions were performed on the ABI 79005 thermocycler using default cycling conditions previously optimized for these assays. Reactions were performed in duplicate and average CT values, if they agreed within 0.4 cycles, were used to calculate absolute quantity. Three runs of RT PCR were performed with overlapping samples in each run to allow normalization of the data. Not all samples were subjected to every assay depending upon sample quantity. Of Note: Sample #4 does not have an H19 transcription value, as on duplicate plating for rtPCR, the CT values did not agree within .4 cycles. Furthermore, samples 15–19 were the final rtPCR of the three runs performed and due to sample scarcity and the need to construct standard curves from pooled samples, only CTCF and BORIS rtPCR’s were performed.

### Western Analysis

**Fig 3:**
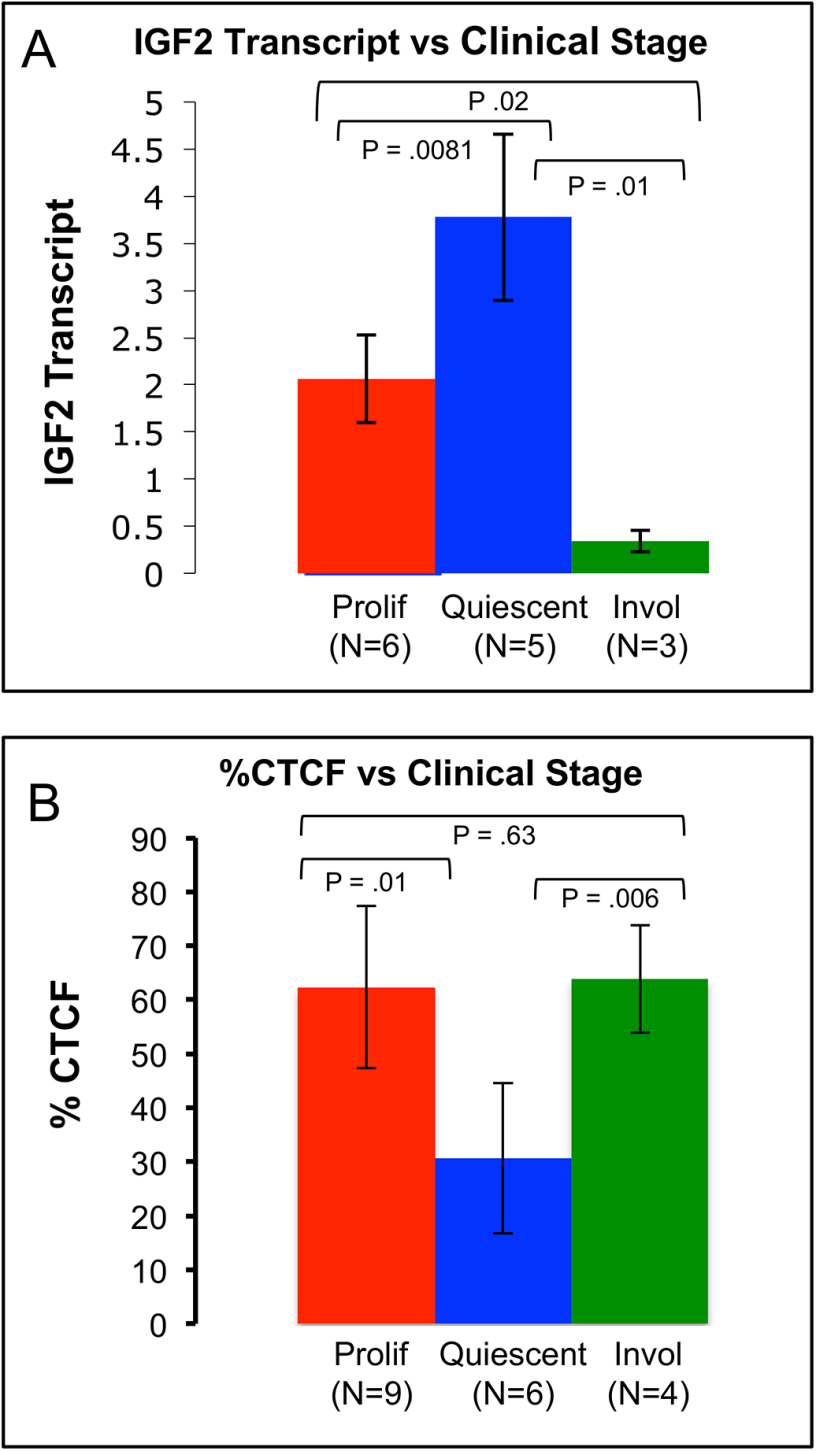
IGF2 Transcription by Clinical Stage Shows an Inverse Relationship to the %CTCF of Identical Stage. **Fig 3A:** IGF2 mRNA levels were approximately 6X lower in the involuting samples compared to their proliferating counterparts. Proliferating vs. involuted p = .02, Proliferating vs. quiescent p = .0081, Quiescent vs. involuting p = .01. Wilcoxian Rank Sum Test. Error bars represent standard deviation. **Fig 3B:** %CTCF changes significantly according to clinical stage. Prolif vs. quiescent p = .01, quiescent vs. invol p = .006, prolif vs. invol p = .63, Wilcoxian two sample test. Error bars represent standard deviation. Note: All samples in the IGF2 analysis were included in the %CTCF analysis, with additional samples.

24 samples were subjected to Western analysis. As this process is tissue intensive, younger samples such as #21 and #23 could only be used for this analysis as insufficient tissue was left for further processing. Other samples were selected biased toward analyzing those samples with transcriptional data in order to compare transcriptional phenomena to translational. However, as the analysis proceeded, presentation gels were constructed to demonstrate key transition points in CTCF and BORIS translation in samples that had not been treated with steroids. Briefly, 50 mg of each sample were processed with a rotary homogenizer in 200ml of RIPA lysis buffer. After centrifugation lysates were created using a standard beta-mercapto-ethanol with SDS. PAGE was performed with 36μg of protein per well in NuPage 10% Bis-Tris precast gels in MOPS buffer at 100 volts. PAGE separated proteins were then transferred for two hours to a PVDF membrane (Bio-Rad) in a standard transfer buffer at 100mAmps. Anti BORIS antibody (Abcam 18337) was used at 1/5000 dilution in TBST with 5% cows milk overnight. Two concentrations of anti-CTCF were used—1:10,000 and 1:5,000—to better visualize late and early rises in CTCF protein (see Fig 3 legend for details.) As anti-CTCF and anti-BORIS were both rabbit polyclonal antibodies they could be visualized simultaneously on the same film following incubation with the anti-rabbit secondary conjugated to horseradish peroxidase and ECL treatment. Images were then scanned and adjusted for brightness and contrast in Adobe Photoshop.

### Bisulfite Methylation Analysis Using Quantitative Pyrosequencing

This method was first described by Grunau *et al*[19] and Dupont *et al*[20]. Protocols specific for each assay in this study are available in S1 Supplementary Information or upon request. Incomplete bisulfite conversion was detected by designing amplicons that contained at least 1 unmethylated cytosine. Primer bias was controlled for by establishing methylation curves of 100% methylated DNA titrated against known amounts of whole genome amplified PCR products that, by definition, are unmethylated. These methylation curves allow experimental samples to be calibrated against known standards. Primers for Exon 9, the H19 promoter and CTCFBS6 as well as the bisulfite-converted sequences they amplify are available in S1 Supplementary Information. The presence of an A/G polymorphism, approximately 130 base pairs downstream of CTCF BS6 leads to primer bias and distorts the absolute methylation values of CTCF BS6, Tost *et al* [21]. See S1 Supplementary Information for details.

### Deducing Parental Contributions of Alleles at CTCF BS6

All samples were subjected to direct sequencing of CTCF BS6 containing the polymorphism rs10732516. Primer design and reaction conditions are available in S1 Supplementary Information. The DNA samples were subjected in parallel to methylation sensitive pyrosequencing of the same polymorphism; please see the section titled “Specimen Collection” for further details. Comparing these results allows each parental contribution to be deduced, see Fig 1 for full details.

### Genomic Southern Analysis for the H19 Promoter

Eighteen were analyzed at a CLIA certified molecular diagnostics laboratory where this assay is performed as a clinical test for Beckwith-Wiedemann Syndrome. The assay is originally described by Debaun *et al*[11]. Norms for this test were previously established with 30 normal controls at 55% methylation with a standard deviation of 5%. All samples were run with a normal and Beckwith-Wiedemann control. The assay exploits a CCCGGG site in the H19 promoter that is cut by the methylation sensitive restriction enzyme *Pst1*.

### Statistical Analysis

Descriptive statistics were used to present patient characteristics. We evaluated the difference in expression of IGF2 transcript across the three developmental stages of IH using a Kruskal-Wallis test. To evaluate if the relative amount of CTCF compared to BORIS transcript changes predictably over time, change point analysis was performed. Change point analysis indicates the likelihood that a change in transcript expression occurred in the sample population by confidence level and a confidence interval regarding when those changes occur. The %CTCF [CTCF/(CTCF + BORIS) x100] was used to develop a change point model that was then compared against clinical staging and IGF2 expression in the sample population. A full explanation of the methods used, as well as a shareware change-point analyzer is presented as an online resource: Taylor, Wayne A. (2000), "Change-Point Analysis: A Powerful New Tool For Detecting Changes," (http://www.variation.com/cpa/tech/changepoint.html.)

To evaluate the association of IGF2 transcript and the relative amounts of CTCF, a linear regression model was fitted, with the %CTCF and age as covariates. The correlation and partial correlation were also calculated. Partial correlations indicate what percentage of variance in IGF2 can be explained by CTCF% alone. Analysis of the covariance model (ANCOVA) was fitted to examine if the correlation between the IGF2 transcript and the difference between CTCF and BORIS varied by the paternal genotype at CTCF BS6, once adjusted by age.

## Results

### Master Data Table

In total, 40 samples were analyzed on a molecular basis. A description of basic demographics, with genotypes at CTCF BS6 and transcript expression values for IGF2, H19, CTCF and BORIS with correlative methylation data was compiled. Please see Fig 1: Master Data Table for details. This table can be utilized to confirm any statistical analysis presented in this study.

### Expression of IGF2, CTCF and BORIS

IGF2 transcription differed significantly by clinical stages (p<0.0001, Kruskal-Wallis test). Plateau stage lesions expressed significantly higher levels of IGF2 than proliferating (p = 0.0081, Wilcoxon rank sum test) and involuted samples (p = 0.02). Involuted hemangiomas expressed the lowest levels of IGF2, approximately 6X lower than their proliferating counterparts (p = 0.01). (See Fig 3A)

**Fig 4:**
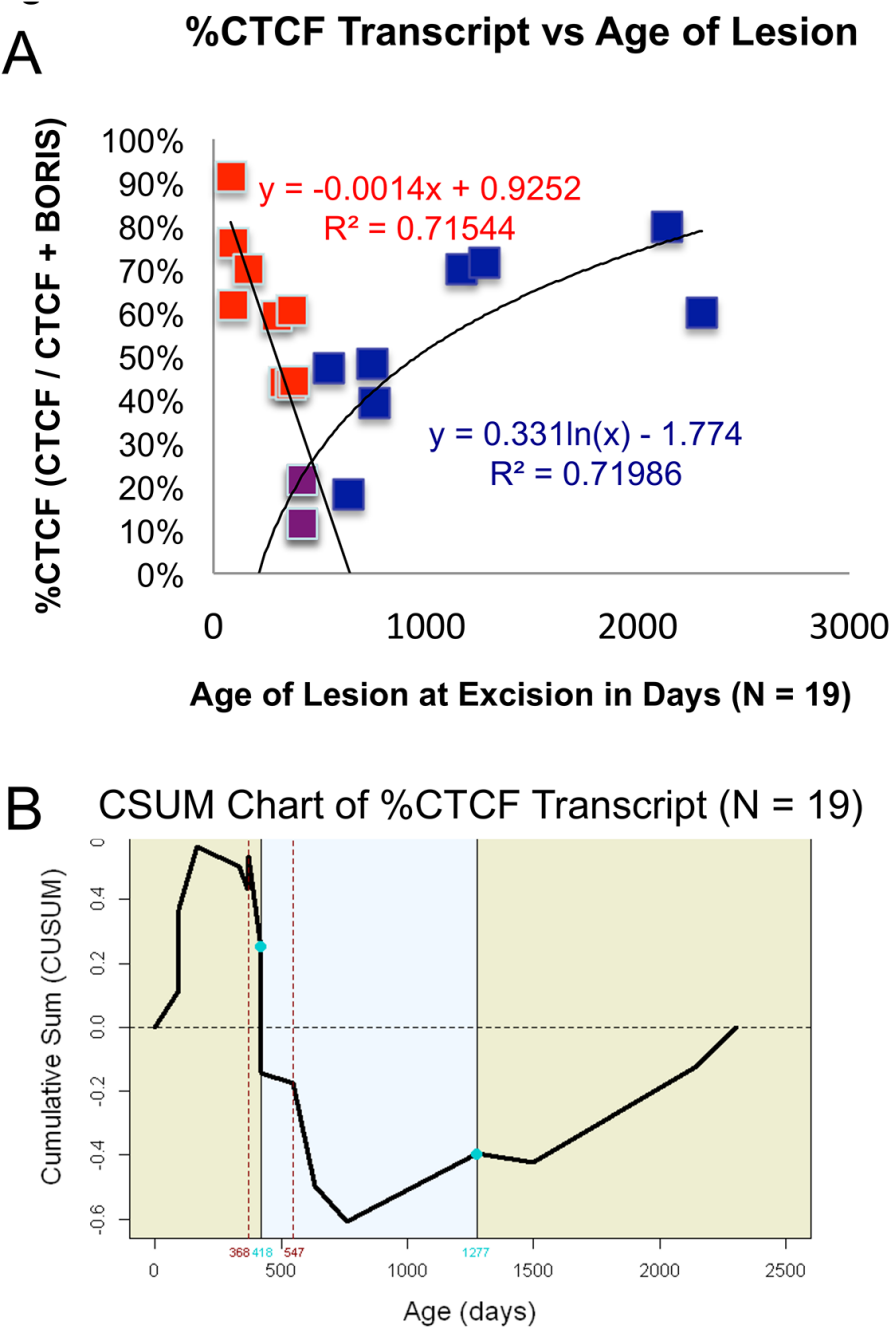
Analyzing the Percentage of CTCF Compared to Total CTCF and BORIS Transcript. (A) Using the two samples with the lowest %CTCF (420 and 418 days as common points (Purple)) two curves with high correlation to age can be appreciated. %CTCF steadily decreases as lesions age until approximately 400 days (red and purple points), then CTCF once again increases compared to BORIS (purple and blue points). This roughly correlates with the transition from proliferative to quiescent lesions. (B) CSUM of %CTCF demonstrates statistically significant variation about the mean of %CTCF according to age. For a full explanation of the CSUM data and commensurate change point analysis, please see S2 Supplementary Information and the online reference in the methods section.

**Fig 5:**
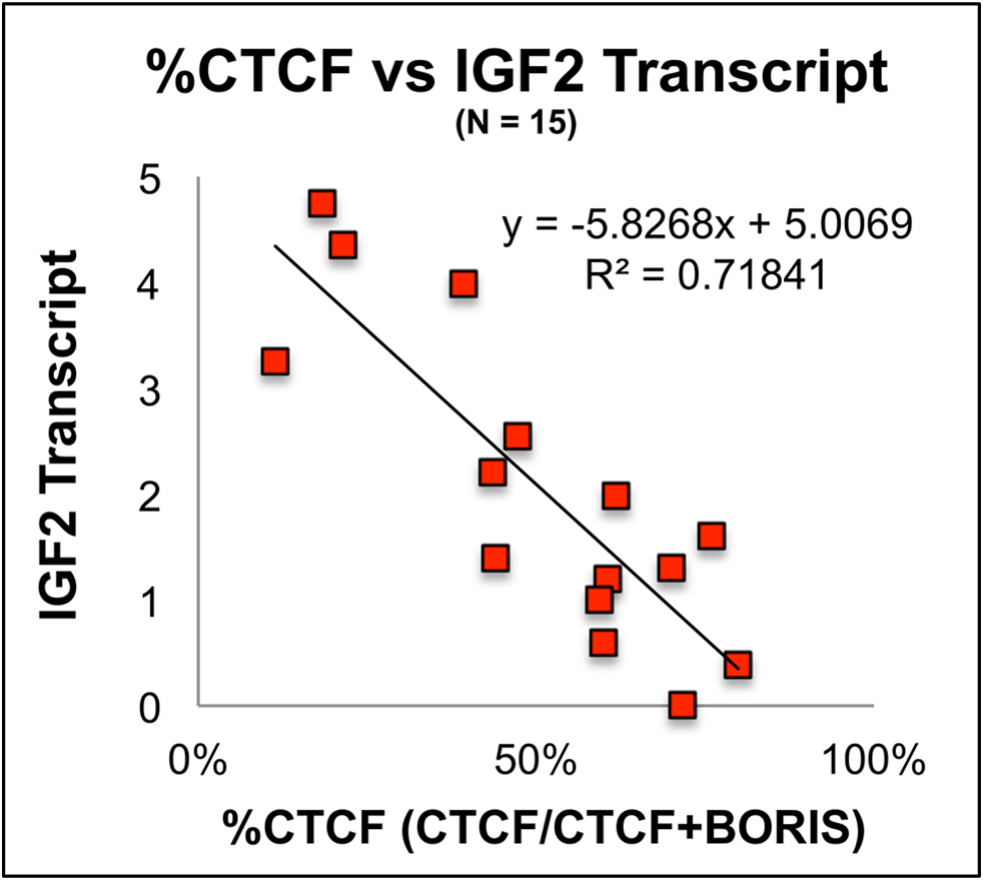
IGF2 Transcript Levels Correlate Inversely With The Percentage of CTCF Compared to Total CTCF + BORIS. To our knowledge, these data represent the first demonstration of the potentially antagonistic effects of CTCF and BORIS on a target gene’s transcription through a continuous curve. P = .0004 ANCOVA Model. Age effect was not significant in the model p = .241. The %CTCF is a stronger statistical predictor of IGF2 expression than BORIS alone .0004 vs. .0028 respectively. (N = 15)

To potentially explain the changes in IGF2 transcription, quantitative RT-PCR was performed for CTCF and BORIS. CTCF and BORIS are co-expressed in all samples. However, the percentage of CTCF transcript compared to total CTCF and BORIS in a given sample [CTCF/(CTCF + BORIS) x 100] varied significantly over developmental time (Figs 3B and 4A). This was confirmed by a change point model (Fig 4B and S2 Supplementary Information): the Y-axis is the cumulative sum (CUSUM) of the differences between %CTCF and the average value of %CTCF. A segment of the CUSUM chart with an upward slope indicates a period where the values tend to be above the overall average. Likewise, a segment with a downward slope indicates a period of time where the values tend to be below the overall average. Based on this analysis, two change points, one estimated at 418 days and the other at 1277 days, were detected. Prior to approximately 418 days (90% CI: 368–547 days), the value of %CTCF tends to maintain a higher level with an average value in this stage equal to 59%. In the second stage (418–1277 days), the level of %CTCF is low with an average 28%. After the second change point at approximately 1277days (90% CI: 760–1500 days), %CTCF has recovered to a high level again with the average 66%. See S2 Supplementary Information for a bar graph analysis of the change point model of %CTCF expression. This result is highly similar to the results obtained by separating samples according to clinical stage (S3 Supplementary Information) %CTCF transcription in IH varies according to clinical stage (Fig 3B) Furthermore, the %CTCF varied inversely with IGF2 transcription (compare Fig 3A and 3B) These two graphs clearly demonstrate that a higher %CTCF corresponds with lower levels of IGF2 expression. Moreover, we detected a strong positive correlation between BORIS and IGF2 transcription (p = 0.0028). Though CTCF alone does not significantly correlate with IGF2 transcript levels, taking both CTCF and BORIS into account using %CTCF is the strongest predictor of IGF2 mRNA expression p = .0004 (Fig 5)

**Fig 6:**
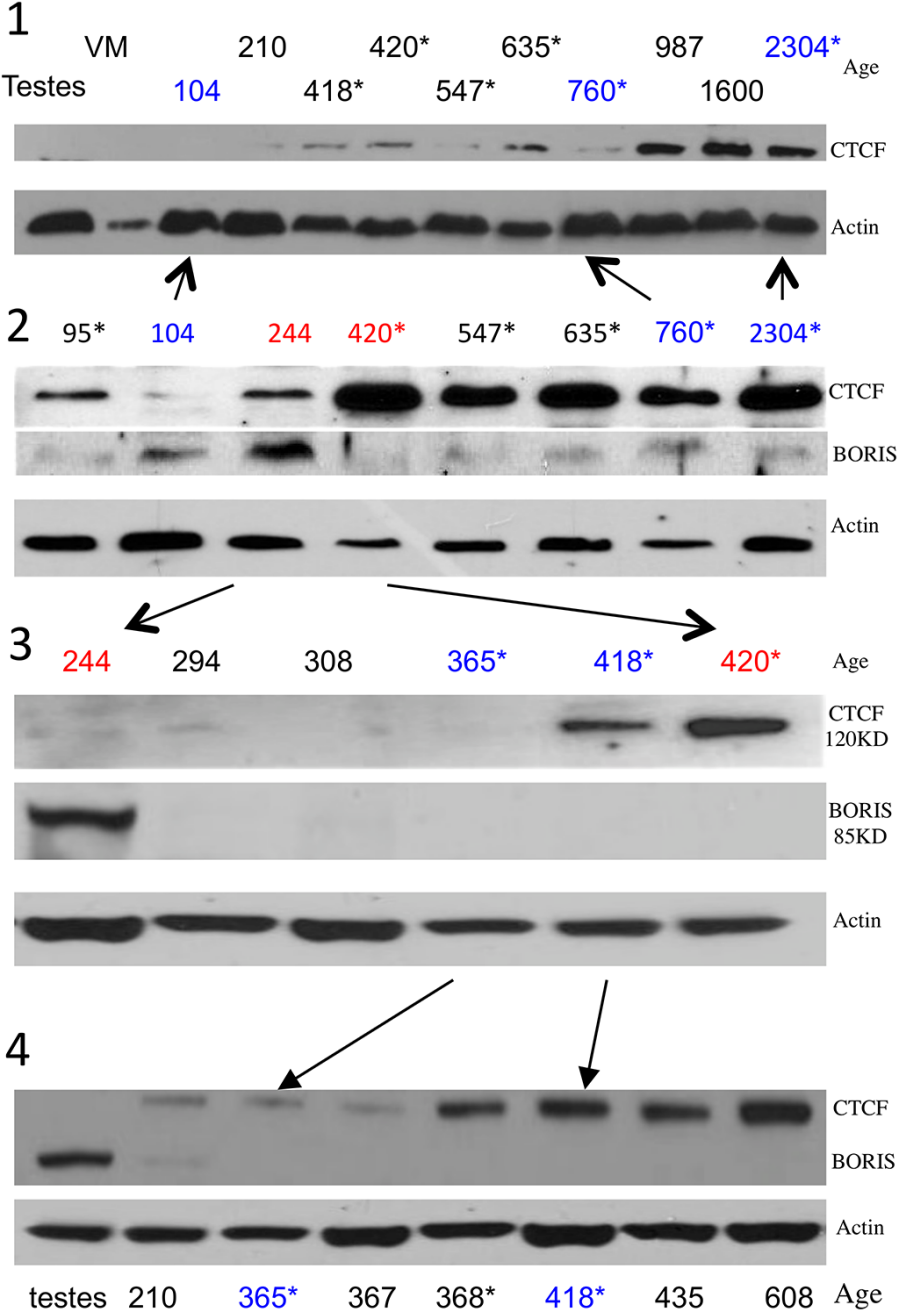
Western Analysis of 24 IH Samples Via 5 Independent Western Blots. Twenty samples with 4 blots depicted, demonstrates 4 Stages of CTCF and BORIS Expression. 6–1: A low concentration of anti-CTCF (1:10,000) demonstrates the complete spectrum of CTCF expression with increases early (210 to 418) and late (760 to 987) in protein expression. (Note, a testes negative control was included as well as a venous malformation denoted as “VM.”) 6–2 through 6–4 were probed with 1:5000 concentration of anti-CTCF that more clearly demonstrates the early rise in CTCF that occurs after 367 days. 6–2 suggests an early increase in BORIS with precipitous downregulation after 244 days. 6–3 and 6–4 expand this critical age range demonstrating a period from 244 days to 367 where BORIS is downregulated but CTCF is not yet upregulated. Note: samples marked with an asterisk were also subjected to CTCF and BORIS rtPCR.

**Fig 7:**
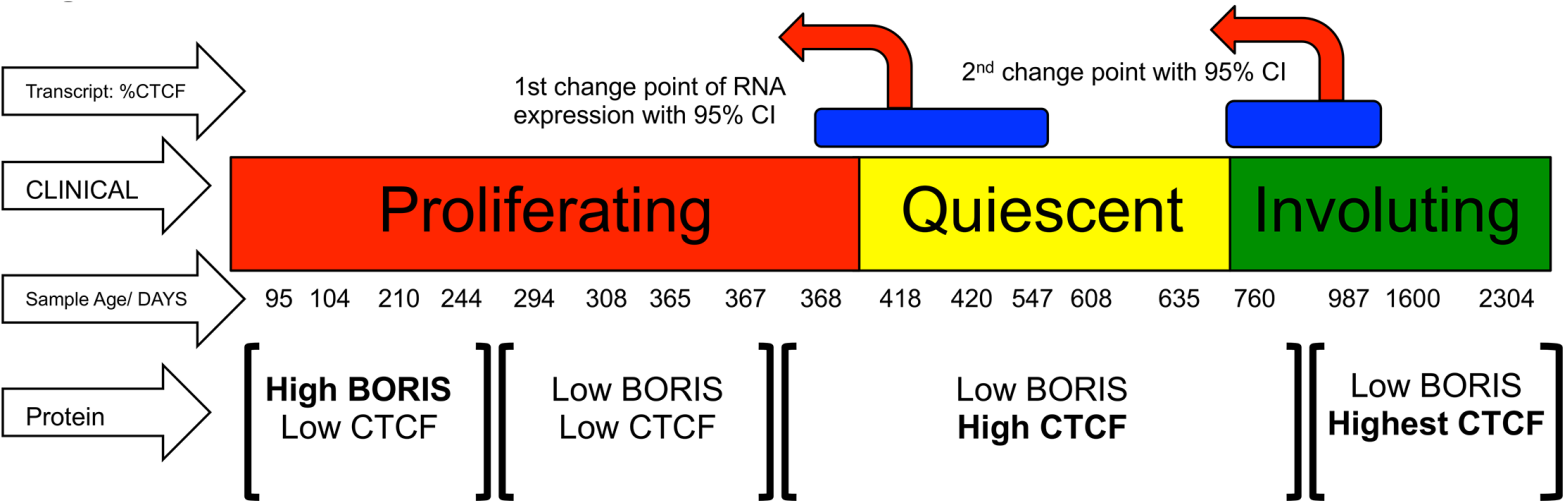
Schematic Integrating CTCF and BORIS Expression Via Both Transcript and Protein with Clinical stage. The Western analysis suggests 4 stages of CTCF and BORIS (see Fig 3, panels 1 to 4) each stage leading to higher levels of CTCF expression relative to BORIS. These interval changes in protein expression closely correlate with clinical stage. Furthermore, blue bars represent the 95% confidence intervals of the two change points identified by quantitative rtPCR. These data show remarkable agreement reinforcing the idea that relative CTCF and BORIS expression levels closely mirror the clinical stage of the lesions tested. Of note, the CTCF and BORIS protein data also suggest a molecular basis for a late proliferative stage.

Western analysis of CTCF and BORIS confirms and expands upon the transcript data (Fig 6) As expected in proliferating lesions, BORIS transcript and protein levels steadily rise in early stage samples (Fig 4A transcript data, Fig 6–2 Western Analysis.) Furthermore, during the transition from quiescent to involuting samples, CTCF mRNA and protein increase compared to BORIS (Figs 3B, 4A and 4B transcript, and Fig 6–1 protein.) Thus, the western and transcriptional data globally confirm one another at the endpoints of IH development. However, the protein data suggests a third change in CTCF and BORIS levels that the transcript change point analysis does not. This third proteomic change appears to take place at the late proliferating to early quiescent phase. It coincides with the so-called late proliferative stage in IH that is suggested by clinicians but not universally accepted. To our knowledge, these data provide the first molecular support for what was previously a clinical category: the late proliferative stage of IH growth. The relative expression of CTCF and BORIS via both transcript and protein levels, is predictive of clinical stage and IGF2 expression. See Fig 7 for details.

### The CTCF to BORIS Transcript Difference (C-B) Predicts IGF2 Transcription According to the Paternal Allele at CTCF BS6

**Fig 8:**
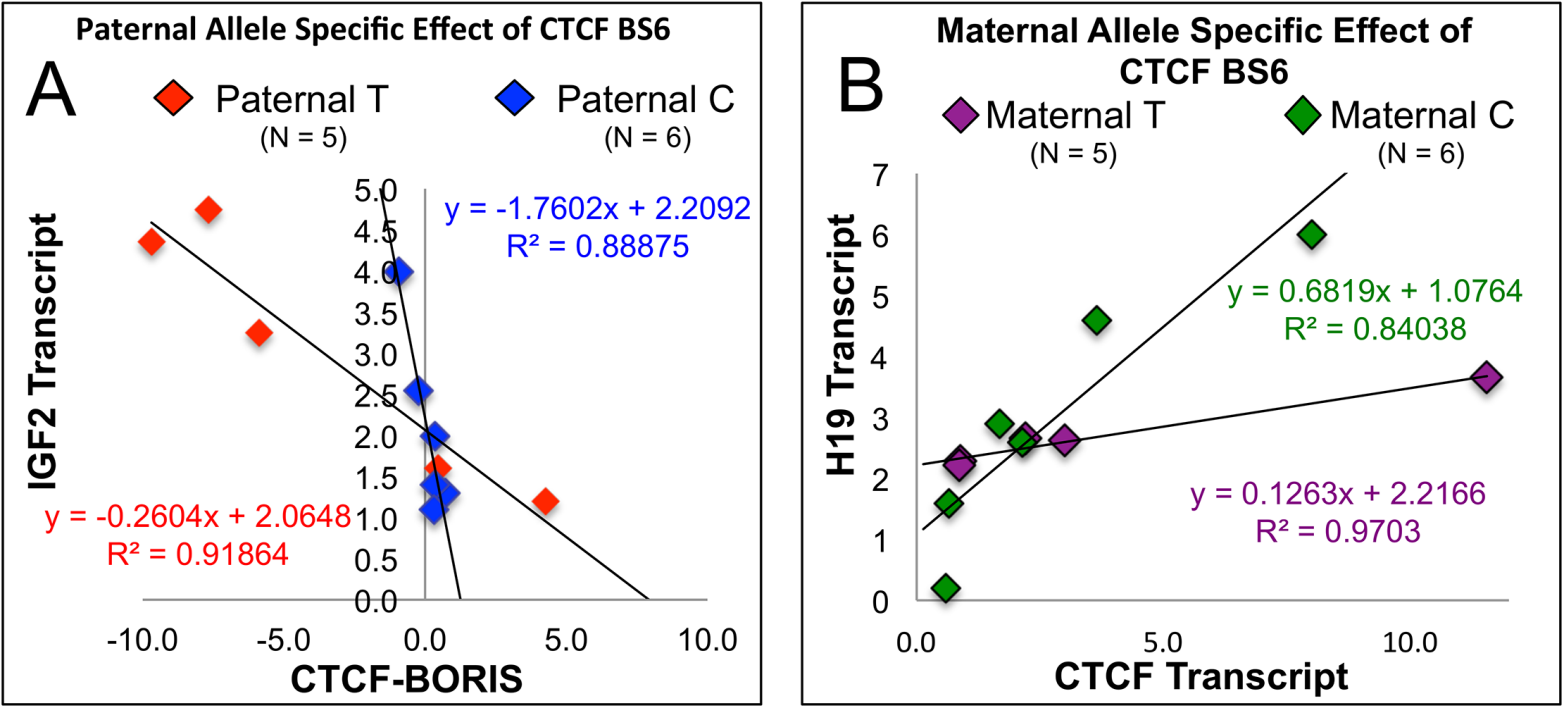
Parent of Origin Specific Effects of CTCF BS6 on IGF2 and H19 Transcription. **Fig 8A:** The antagonism of CTCF and BORIS relative to IGF2 production is demonstrated by calculating the difference between the two. Here IGF2 transcription correlates positively with higher relative levels of BORIS compared to CTCF. However, two curves are apparent. These are separated by the paternally contributed allele at CTCF BS6. Paternal C samples lay on a steeper curve than the paternal T counterparts (P = .05.) Note: Involuted samples were not considered in the analysis as they make similarly low levels of IGF2 regardless of paternal genotype. There is no clear relationship when samples are sorted by maternal genotype (S4 Supplementary Information). **Fig 8B:** H19 positively correlates with CTCF transcript levels alone according to maternal genotype: Maternal C samples lie on a steeper curve than their maternal T counterparts (p = 0.0129, ANCOVA). There is no clear relationship when samples are sorted by paternal genotype (S4 Supplementary Information).

This study utilizes existing technologies: Direct sequencing of the known polymorphism of CTCF BS6 (rs10732516) with a previously described methylation assay for CTCF BS6 [21]. Applying these two assays in a novel manner (see Fig 2) allowed us to deduce both maternal and paternal contributions to CTCF BS6—which will be referred to as the maternal and paternal contribution (Fig 8.) This paternal contribution likely has significant effects on IGF2 production as it relates to CTCF and BORIS.

IGF2 mRNA is demonstrated to be inversely related to CTCF and positively correlated to BORIS transcripts when plotted against %CTCF (Fig 5). By using the difference between CTCF and BORIS (C-B) rather than the %CTCF this relationship can be differentiated by the paternally contributed allele at CTCF BS6 (Fig 8A) The paternal allele of a common C/T polymorphism within CTCF BS6 (rs10732516) corresponds with two strikingly different CTCF-BORIS vs. IGF2 curves. After age adjustment, the effect of CTCF-BORIS on IGF2 transcription was significantly different between patients bearing different paternal alleles (p = 0.05, ANCOVA model) and there is a strong correlation between IGF2 expression and CTCF-BORIS (p = 0.0007). The samples bearing a paternal C allele, appear to demonstrate a six fold steeper slope of IGF2 mRNA relative to the CTCF–BORIS difference, compared to their paternal T bearing counterparts. This allele was identified in both tissue and patient matched control blood. Of note, heterozygote analysis revealed no clear relationship regarding these factors according to the maternal allele (p = 0.95, ANCOVA model, see S4B Supplementary Information). It remains a possibility that the paternal allele effect may be steroid treatment driven as more samples with the paternal T allele were treated with steroids than the paternal C allele. This potential bias was investigated with an odds ratio calculation sorting steroid treatment according to paternal genotype. The odds ratio suggested that paternal T samples were more likely to be treated with steroids but this result did not reach statistical significance (See S4D and S4E Supplementary Information.) As the allele specific analysis was done on only proliferative samples the odds ratio calculation was performed twice, once including involuted samples and once to their exclusion. However, we acknowledge that the odds ratio suggested a potential steroid treatment bias in paternal T samples, which may have become significant in a larger patient cohort (See S4D and S4E Supplementary Information for details.) Lastly, IGF2 expression in IH was mono-allelic in all 5 informative heterozygotes tested for a known IGF2 polymorphism in exon 9[9]. IGF2 imprinting status appears to be maintained despite BORIS expression (S5 Supplementary Information.)

### H19 Transcript Levels Correlate Positively with CTCF mRNA According to the Maternally Contributed Allele at CTCFBS6

After age adjustment, CTCF transcript levels alone correlated positively with H19 transcription but only when separated by maternal genotype ((p = 0.0150, Fig 8B) Moreover, this positive correlation is significantly different among patients with different maternal alleles (p = 0.0129, ANCOVA). The correlation between CTCF and H19 transcription is stronger in patients bearing a maternal C allele compared to their maternal T counterparts. There were no identifiable relationships between H19 transcription and either the paternal genotype at CTCF BS6 or BORIS mRNA, p = .8 ANCOVA (S4A Supplementary Information.) There also appeared to be no relationship between H19 expression and clinical stage of the hemangioma (S4C Supplementary Information).

It remains a possibility that the maternal allele effect may be steroid treatment driven as more samples with the maternal T allele were treated with steroids than the maternal C allele. This potential bias was investigated with an odds ratio calculation sorting steroid treatment according to maternal genotype. The odds ratio suggested that maternal T samples were more likely to be treated with steroids but this result did not reach statistical significance (See S4F Supplementary Information.) However, we do acknowledge that this odds ratio may have become statistically significant in a larger sample size; the effect was not great enough to significantly bias our sample size.

### CTCF Transcript Levels Alone Correlate with Demethylation of the H19 Promoter

**Fig 9:**
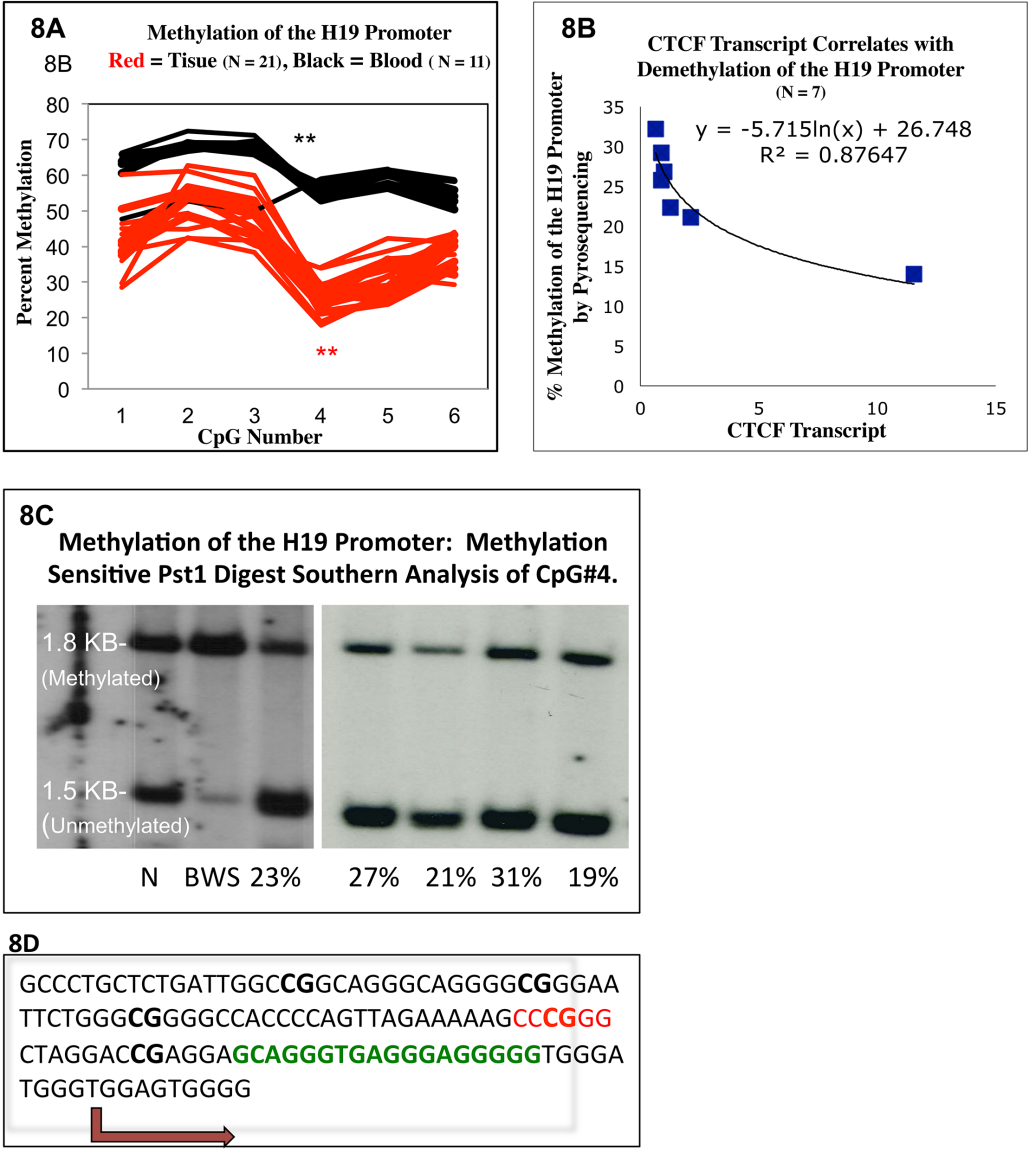
CTCF Expression and H19 Promoter Methylation. **Fig 9A:** Increased CTCF transcript level correlates with demethylation of the H19 Promoter. Those samples with the highest CTCF expression were the least methylated ranging from 34% to 14%. However, demethylation of the H19 promoter did not correlate strictly with H19 transcript expression (S6 Supplementary Information). **Fig 9B and 9C**—The H19 promoter (see Fig 9D) is hypomethylated, demonstrated by bisultife converted pyrosequencing (9B) and methylation sensitive restriction digest with southern hybridization (9C.) 25 IH samples, and 13 matched blood controls were subjected to bisulfite converted pyrosequencing. 13 IH samples and 13 matched blood controls were subjected to southern analysis with methylation sensitive Pst1 digestion. Two representative gels show, 5 IH samples, a Beckwith-Weidman positive control and a 50% methylated normal control. **Fig 9D**: sequence showing the H19 promoter—CpG#4 of the bisulfite sequencing test corresponds to the CCCGGG Pst1 digestion site of the Southern analysis. Other CpG’s tested are in bold. This CpG is in close proximity to the transcription start site of H19 (blue arrow) and an overlapping putative CTCF binding site identified by positional weight matrix analysis.

All IH samples tested demonstrated significant hypomethylation at the H19 promoter compared to matched patient blood controls (Fig 9A) This was demonstrated with bisulfite specific pyrosequencing (Fig 9B) and confirmed with methylation sensitive enzyme digest and southern analysis (Fig 9C) As pyrosequencing is quantitative, we correlated these data with CTCF and BORIS expression in matched samples. Decreased promoter methylation correlated with higher levels of CTCF (p = 0.015; simple regression. Fig 9B). Yet, this finding may be subject to confounders as it is not statistically significant after age adjustment (p = 0.17). As the H19 promoter is paternally methylated and maternally demethylated, a methylation level below 50% would entail demethylation of the paternal allele in at least a subset of cells within a given sample. However, no bi-allelic expression of H19 could be detected in 5 heterozygote samples (S5 Supplementary Information.) Furthermore, H19 promoter demethylation did not strongly correlate with H19 expression (S6 Supplementary Information.)

### Multiple Imprinted Sites Within the IGF2/H19 Locus are Abnormally Methylated in IH Compared to Matched Control Blood

It remains a formal possibility that the normalization of CTCF to BORIS ratios, as well as decreased IGF2 transcription, in involuting and involuted samples is not due to an intracellular phenomena but rather the incremental replacement of abnormal IH tissue (vascular stroma) with normal tissue (fat.) Thus, the results we are presenting are the product of tissue heterogeneity. We acknowledge that IH lesions transform from a vascular tumor into a fibrofatty residuum; therefore, the transitional phases are by definition composed of heterogeneous cell populations. However, we see no evidence that the fibrofatty residuum of an involuted IH represents “normal” tissue. To the contrary, many of the methylation abnormalities discovered by this study are either stable or progressive from early to late clinical stages. For instance, the H19 promoter is significantly demethylated in all IH samples (see Fig 9A–9C). However, the demethylation is progressive with age (S7 Supplementary Information.) Furthermore, we also found focal demethylation at Exon 9 and hypermethylation at DMR0, deviating from the expected 50% for these known imprinted sites. Both findings remained consistent in all IH clinical types and were age independent (S8 and S9 Supplementary Information for details.) If IH tissues were being replaced by normal fat we would expect the methylation abnormalities demonstrated in this work to normalize, not remain constant or even progress with age. Given this argument, the simplest explanation for the methylation data is that IH tissue begins as epigenetically abnormal vascular stroma and transforms into epigenetically abnormal fat or is at least replaced by the like.

### CTCF BS6 Genotypes Correlate With Clinical Outcomes

**Fig 10:**
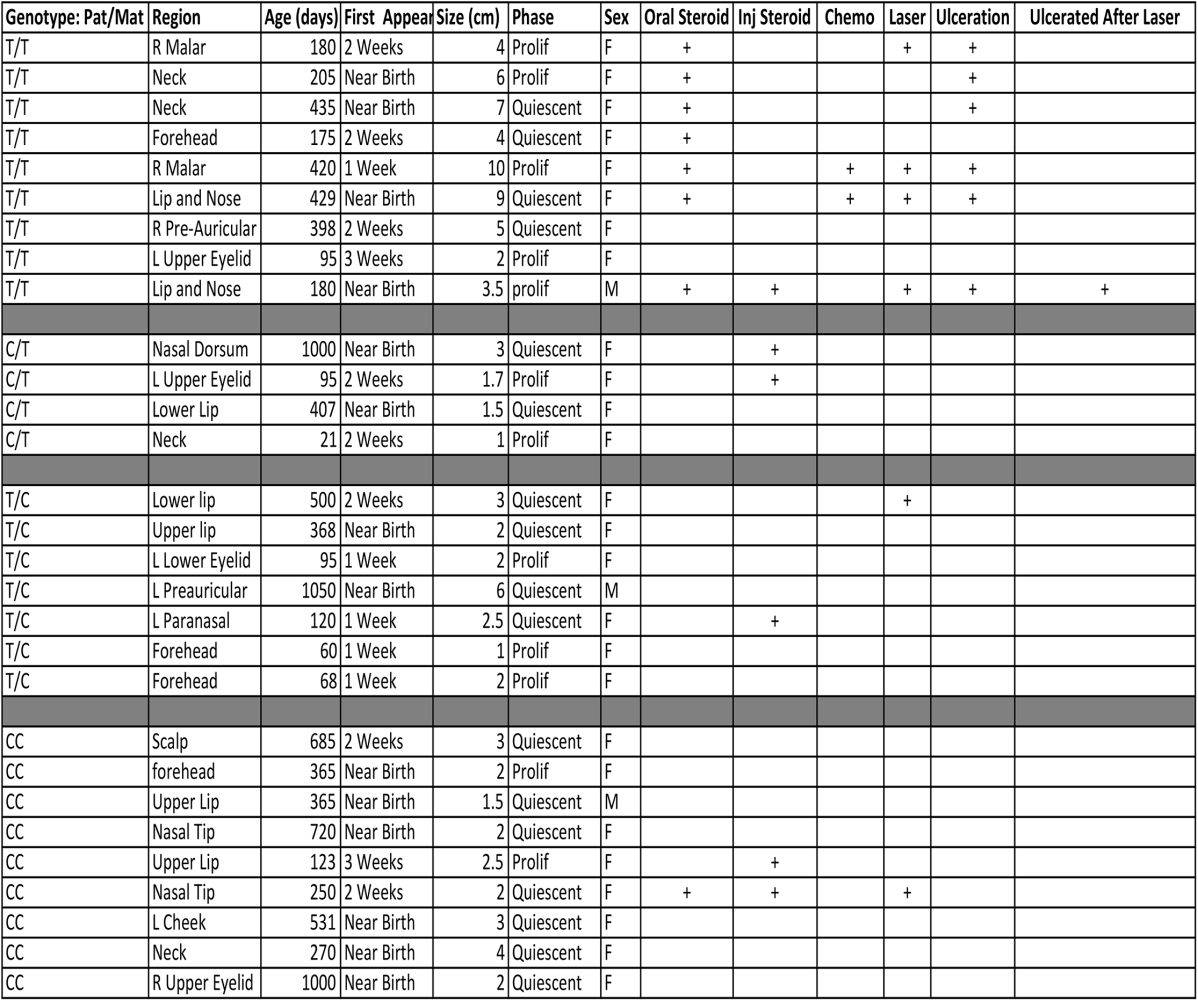
Summary of clinical data. Retrospectively collected results with associated descriptive information.

**Fig 11:**
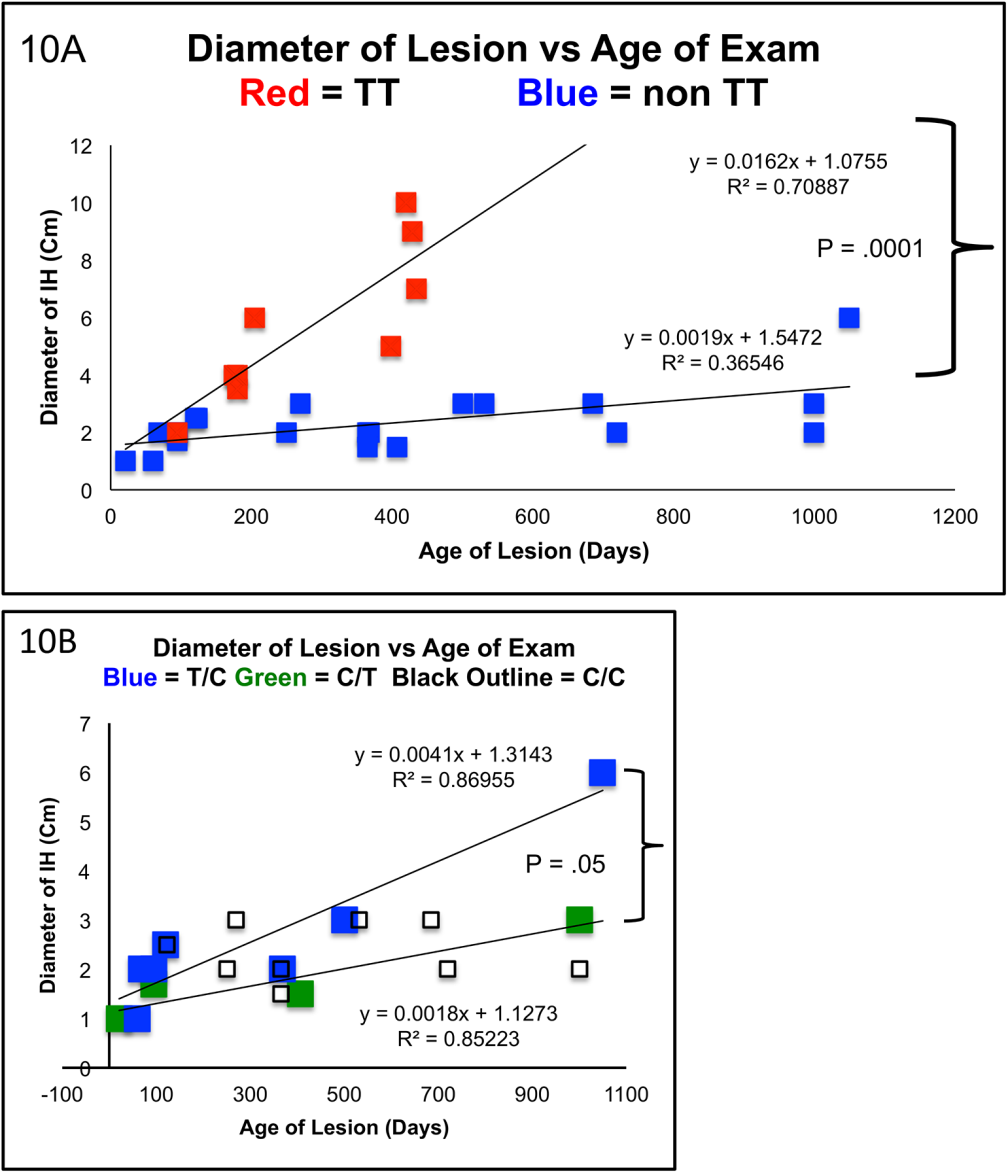
Clinical Correlation of Hemangioma Growth Rates with Parental Contributions to CTCF BS6. **Fig 11A:** This retrospective analysis of 29 samples, 9 TT, 20 non TT, demonstrates significantly distinct growth curves over a large age range. The ANCOVA model has identified age as a predictor of size p = .0007. The association between tumor size and age is significantly different among the genotypes of TT, C/T, T/C and CC p < .0001. *Of Note the paternal contribution is presented first and the maternal is second*. The interaction terms of parentally specific genotypes allowed us to test if the slopes of the curves between tumor size and age are different among the genotypes. This analysis indicted that an increase in 1 day of age is associated with .016cm of growth in the TT group. This is significantly higher than the non TT group p = .0019. **Fig 11B:** Growth analysis focusing on the “non T/T” group. Each non TT growth curve varied independently and significantly from the TT samples (CC vs. TT: P<0.0001, CT vs. TT: P<0.0008, TC vs. TT: P = 0.0025). Furthermore, these data suggest parent of origin specific effects as those samples with identical genotypes but opposite parental contributions displayed statistically significant differences in growth curves. The paternal T/maternal C genotype grew at approximately twice the rate as their paternal C/ maternal T carrying counterparts (p = .05). The homozygous C group appeared to have a roughly flat growth rate between the heterozygotes and did not significantly vary with either heterozygote group (CC vs. C/T p = .99, CC vs. T/C p = .74)

Mechanisms aside, parent of origin specific effects are demonstrated at the molecular level regarding expression patterns of both IGF2 and H19. However, the question remains whether these molecular phenotypes may translate into clinically significant growth patterns. Fig 10 is a complete table of all patients participating in this retrospective clinical study. For details of subject inclusion please see methods section. Size of the lesion as well as the date of examination was included with relevant clinical information such as sex, medical treatments utilized and presence of ulceration during clinical course. Each patient was sorted according to CTCF Binding Site Six Genotype and paternal contribution for heterozygotes. By plotting the size of IH lesions against the CTCF BS6 genotypes, four distinct growth curves emerge (Fig 11A and 11B) The association between tumor size and age (days) are significantly different among these four genotypes (separating heterozygotes by their respective parental contributions) CC, C/T, T/C, TT (p = 0.0162, ACOVA.) The most impressive growth phenotype was exhibited by homozygous T samples reaching an average of 7.8cm before excision (Fig 11A) Comparing the TT group against all non-TT subjects, the difference in lesion size, increased significantly with age (p = 0.0001, ANCOVA). Thus in this study, TT lesions grew more rapidly than non-TT genotypes. In fact, each growth curve—separated by maternal and paternal genotype—varied independently and significantly from the TT samples (CC vs. TT: P<0.0001, CT vs. TT: P<0.0008, TC vs. TT: P = 0.0025). Although genotype appears to have no effect at approximately 100 days, after three months, lesions begin to distinguish themselves suggesting distinct growth velocities. Furthermore, these data suggest parent of origin specific effects as those samples with identical genotypes but opposite parental contributions displayed statistically significant differences in growth curves. Namely, those lesions carrying the paternal T /maternal C genotype grew at approximately twice the rate as their paternal C/ maternal T carrying counterparts p = .05 (Fig 11B) Furthermore, each heterozygote growth curve varied by age with high correlation of r squared above .85. However, it must be emphasized that sample size is relatively low (particularly in the C paternal/T maternal group) and the p-value just reached the threshold of significance.

**Fig 12:**
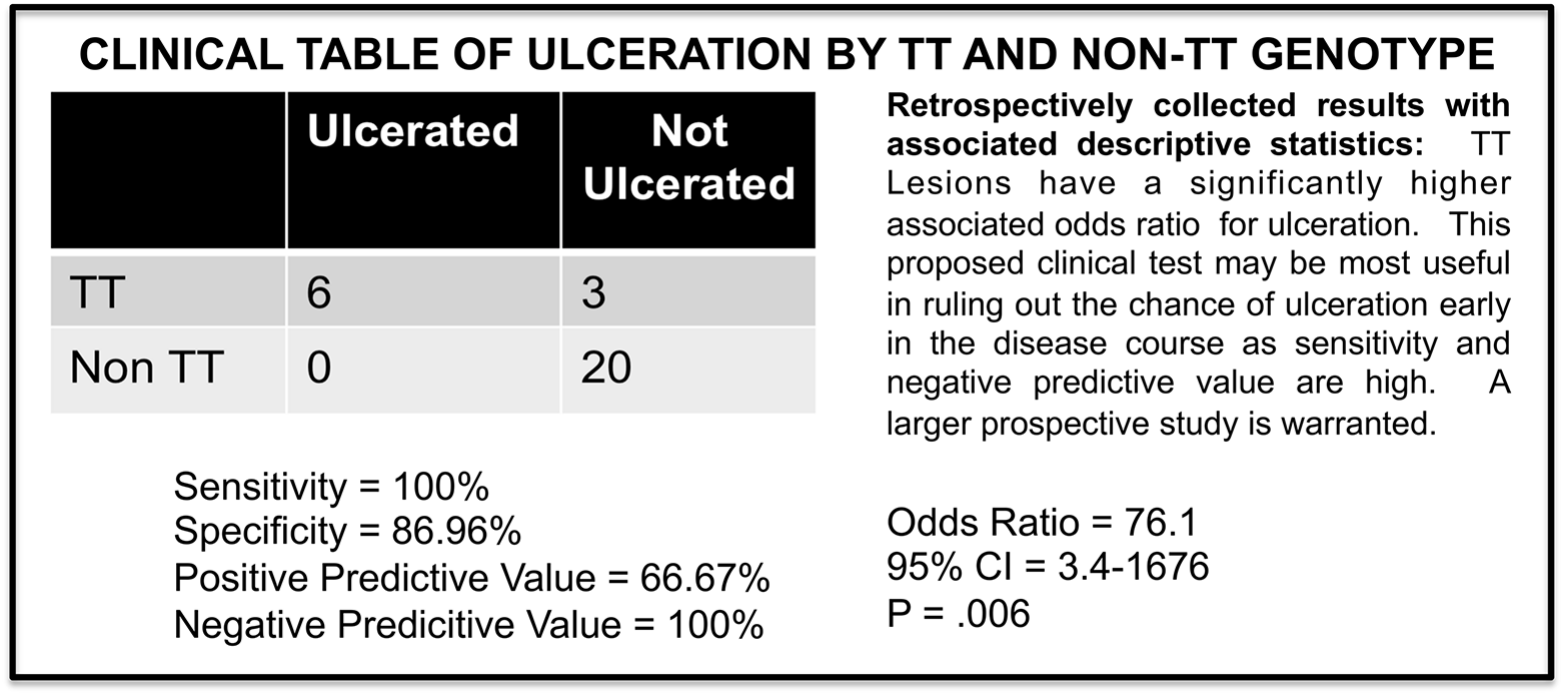
TT Lesions Have a Significantly Higher Associated Odds Ratio for Ulceration. This proposed clinical test could be most useful in ruling out the chance of ulceration early in the disease course as sensitivity and negative predictive value are high. A larger prospective study is warranted.

Lastly, size is a highly significant clinical outcome when studying IH. However, of similar importance is ulceration. Once an IH ulcerates, it is usually painful for the patient and is given to bleeding which can be clinically significant. Ulceration is usually a marker of rapid disease progression and heralds an escalation of care. This can entail the institution of laser therapy, pharmacologic intervention or surgical excision. Not surprisingly, ulceration alone can prompt surgical treatment regardless of size or location of the lesion. To study the risk of ulceration an odds ratio calculation was performed comparing TT and non-TT lesions. The TT lesions had an odds ratio of 76.1 for ulceration p = .006 (Fig 12) Although this is a small sample cohort we were able to perform preliminary specificity, sensitivity and positive and negative predictive value calculations (Fig 12) These early results suggest the highest clinical usefulness of the proposed test in ruling out potential future ulceration. Although encouraging, these results will need to be corroborated prospectively in a larger cohort.

## Discussion

Hemangiomas are unique within the spectrum of human tumorgenesis. Unlike most highly proliferative lesions, hemangiomas eventually regress instead of undergoing malignant transformation [1,2,6]. The benign nature of IH is surprising given BORIS expression. Primarily, BORIS is considered a cancer/testis gene. Furthermore, the majority of common malignancies express BORIS: 80% of lymphomas, breast, osteosarcoma and melanoma, among others [22–24]. In view of these facts, perhaps the most germane question is not how hemangiomas begin, but rather why they end.

Jones *et al* claim to have identified BORIS expression in multiple normal tissues outside the testes [25]. They found that CTCF was expressed in abundance to BORIS except in the testes where they are comparable, suggesting that CTCF predominance is onco-suppressive. Furthermore, Woloszynska *et al* recently compared CTCF and BORIS quantitatively in human ovarian cancer where they found only stage III and higher disease was BORIS predominant [26]. In IH, CTCF and BORIS are expressed over a broad range. Proliferative samples expressed higher levels of BORIS relative to CTCF and became BORIS predominant near the one-year mark. Intriguingly, despite this apparently oncogenic expression pattern, IH cedes its malignant potential as lesions involute and return to states of CTCF predominance. These changes proceed gradually at the transcript level but are discrete regarding protein expression. In samples where both RT-PCR and Westerns were performed, the return to CTCF predominance is seen first at the protein level and followed by a transcriptional shift. This suggests that a non-malignant profile of BORIS to CTCF is maintained at the post-transcriptional level. As direct CTCF binding to the BORIS promoter is a known repressor of BORIS transcription [27], perhaps the changes of CTCF relative to BORIS protein then trigger a transcriptional shift. The specific mechanism of this shift in translation remains to be investigated, but may have implications beyond benign tumors

CTCF’s pivotal role in maintaining the imprinted regulation of IGF2 and H19 is supported by a decade of research [18,22,28–36]. Experimental evidence suggests that CTCF and BORIS oppose one another functionally with similar DNA binding domains but distinct C and N terminal sequences as well as protein binding partners. The IGF2/H19 locus is a classic example of this potential rivalry. As CTCF binding is necessary to protect maternally unmethylated regulatory regions [16,29,37], ectopic BORIS potentially methylates those same functional sites [38]. In tissues expressing both transcripts, IGF2 transcript levels should reflect a function of both CTCF and BORIS. We have demonstrated this, via ratio or difference, through a continuous curve. To our knowledge, this study represents the first *in vivo* titration curve of the effects of CTCF and BORIS on IGF2 and H19 transcript levels. These data suggest that IH is a powerful potential model system to study the relative effects of CTCF and BORIS across a broad range of levels, at least within the IGF2 locus and perhaps beyond.

A close examination of this CTCF to BORIS titration curve reveals two parent of origin specific phenomena. 1) IGF2 mRNA transcription can be separated according to paternal genotype at CTCF BS6 and is associated with both CTCF and BORIS levels. 2) H19 expression can be separated according to maternal genotype and is strongly associated with increased levels of CTCF alone. Previous studies have found associations between polymorphisms within the IGF2 gene relating to birth weight [39], progression of ovarian[40] and breast cancer[41], and one such study suggested parent of origin effects[39]. Furthermore, a recent study found an association with the maternal T genotype of CTCF BS6 and increased methylation of the maternal chromatid[42]. No such studies however, to our knowledge, relate these data directly to a functional mechanism such as IGF2/H19 expression and none have shown correlation between any phenotype and a polymorphism within or near the key regulatory element CTCF BS6. As for H19 expression, this study suggests that not all imprinted or differentially methylated sites respond to CTCF and BORIS levels in the same way. Recently, Sleutels *et al* have demonstrated, in the mouse, incompletely overlapping DNA binding site sets for CTCF and BORIS, corroborating our observations[43]. Specifically, our data suggest that BORIS does not affect methylation at the H19 promoter while CTCF likely does, recognizing the caveat of age related confounders. Furthermore, H19 expression was positively related to CTCF transcript alone and this correlation improved significantly by separating samples according to the maternal genotype at CTCF BS6. As H19 is transcribed maternally, the maternal genotype effect, and lack of a paternal effect found in this study, agrees with current models. Our data provide further evidence, albeit circumstantial, that the maternal allele of CTCF BS6 interacts with the H19 promoter. Moreover, as each allelic combination is associated with a different regulatory curve of IGF2 and H19, each parent of origin specific genotype also fits within a suggested clinical growth curve.

Regardless of the mechanism, this retrospective study demonstrates that homozygous T samples clearly exhibited the strongest growth phenotype. Interestingly, all allelic combinations appeared similar in size at 3 months. Yet, beyond this juncture, the T/T genotype clearly embarked on an accelerated growth curve compared to other genotypes. Each heterozygote state also appeared to exhibit a distinct growth pattern. Interestingly, the homozygous C samples did not demonstrate a clear growth pattern and were not statistically different from the heterozygote groups. One possibility is that they may follow a distinct but intersecting growth curve with both the heterozygotes. Another possibility is that the CC genotype allows for an individual IH to associate with either of the heterozygote trajectories. Given the CC data, it remains a formal possibility that the T allele is in linkage disequilibrium with a nearby growth enhancing allele; thus, the C/T polymorphism may not be a functional SNP but rather closely linked to one; for instance, a common G/A polymorphism lies 130bp distal to CTCF BS6[21]. However, this polymorphism, like others proximate to CTCF BS6, cannot be easily typed into maternal and paternal contributions using the patient’s DNA alone. Thus, the test we propose, for clinical purposes, is likely to be the most useful. This work would now benefit from a prospective study with more statistical power.

Although this study does not offer explanations for all of the clinical variability observed in IH, it does suggest a genetic marker that may predict some of this variability. This test consistently identified the most high-risk individuals where early intervention before 1–3 months should be considered[7]. Furthermore, this study suggests two other distinct growth patterns from which clinical decisions could be made comparing lesion size to age. Such comparisons may be informative when considering treatment. However, it must be stressed our retrospective study requires prospective confirmation. Furthermore, the study is likely affected by considerable selection bias as all of the children in our study were referred to a surgeon for possible operative management. Every lesion in this study was surgically resected, which is the exception rather than the rule regarding the treatment of most IH. These lesions likely represent the most aggressive forms of IH and we caution extrapolating the results of this study to a non-surgical practice. This concern is underscored by the fact that the growth curves suggested by our data differ from Chang *et al* at the upper limits of age [7]. It must be emphasized that the data for this study was collected before the era of propranolol. In view of that fact, the growth curves generated, despite their limitations, can no longer be obtained. E.g. aggressive lesions are rarely allowed to grow to a diameter of 10 cm. Once an aggressive lesion is identified, it is usually treated promptly with a beta-blocker. As the side effect profile is less than that of steroids, lesions are usually treated earlier and to greater effect. These data represent the best and only information regarding the potential phenotypic effects of CTCF BS6 on IH growth. If taken in proper context, we believe that this clinical study offers a unique and enriching dialogue to the overall literature on IH. Although beta-blocker therapy has improved the management of IH, there is still opportunity for earlier and even more effective interventions. Assuming this work can be validated in a prospective manner, these results offer the possibility of identifying high-risk genotypes not only for prodigious growth but also ulceration. Such a validated test may justify pre-emptive treatment.

Within the gamut of human tumors, IH is unique. Studying the pathogenesis of IH is an opportunity to compare dynamic molecular processes against an equally dynamic but predictable disease course. Central to these findings is that IH can now be classified as the first non-malignant BORIS positive tumor. A natural “dose response” curve of CTCF to BORIS can be used to explore the potential effects of these regulators across a gamut of molecular and clinical phenomena. This analysis revealed possible parent of origin specific effects of CTCF BS6 at both the cellular and clinical levels. This genotype to phenotype analysis offers the first testable model that may explain much of the clinical variation of IH. If validated with prospective work, identifying high-risk genotypes, would obviate the need to wait for lesions to become aggressive and decrease the complications of delay. Furthermore, the generalizability of these results must be explored regarding malignant tumors that are BORIS positive.

## Supporting Information

S1 Supplementary Information Supplemental Methods and Materials (TIF)

S2 Supplementary Information Descriptive Statistics of %CTCF Expression vs Age of lesion Whithin the Study Group—CSUM Model (TIF)

S3 Supplementary Information Descriptive Statistics of %CTCF—Classifying Lesions by Clinical Stage vs Change Point Model Yield Similar Results (TIF)

S4 Supplementary Information Allele Specific Analysis of IGF2 and H19 Expression by Parental Contribution at CTCF BS6. S4A—IGF2 by Maternal Genotype. S4B—H19 by Paternal Genotype. S4D-F—Odds Ratios for Potential Steroid Treatment Bias by CTCF BS6 Parental Contribution.
(TIF)

S5 Supplementary Information Imprinting of IGF2 and H19 Appear to be Maintained in IH for all Age Ranges. (TIF)

S6 Supplementary Information H19 Expression by Promoter Methylation (S6A) and by Clinical Stage (S6B.) (TIF)

S7 Supplementary Information H19 Promoter Methylation Decreases According to Lesion Age. (TIF)

S8 Supplementary Information Abnormal Demethylation of one CpG in IGF2 Exon 9 is Maintained in all IH lesions, Regardless of Age. (TIF)

S9 Supplementary Information Abnormal Hypermethylation of DMR0 is Maintained in all IH Lesions, Regardless of Age. (TIF)
