# Correction: A Common Polymorphism within the IGF2 Imprinting Control Region Is Associated with Parent of Origin Specific Effects in Infantile Hemangiomas

**DOI:** 10.1371/journal.pone.0143806

**Published:** 2015-11-24

**Authors:** The PLOS ONE Staff

The images for Figs 1 and 2 are incorrectly switched. The image that appears as Fig 1 should be Fig 2, and the image that appears as Fig 2 should be Fig 1. The Figure captions appear in the correct order. Please see the corrected Figs 1 and 2 here.

There is an error in the last sentence of the Deducing Parental Contributions of Alleles at CTCF BS6 subsection under Materials and Methods. The correct sentence is: Comparing these results allows each parental contribution to be deduced, see Fig 2 for full details.

The panel labels for Figs 9 and 11 appear incorrectly in the published article. Please see the corrected Figs 9 and 11 here.

The publisher apologizes for the errors.

**Fig 1:**
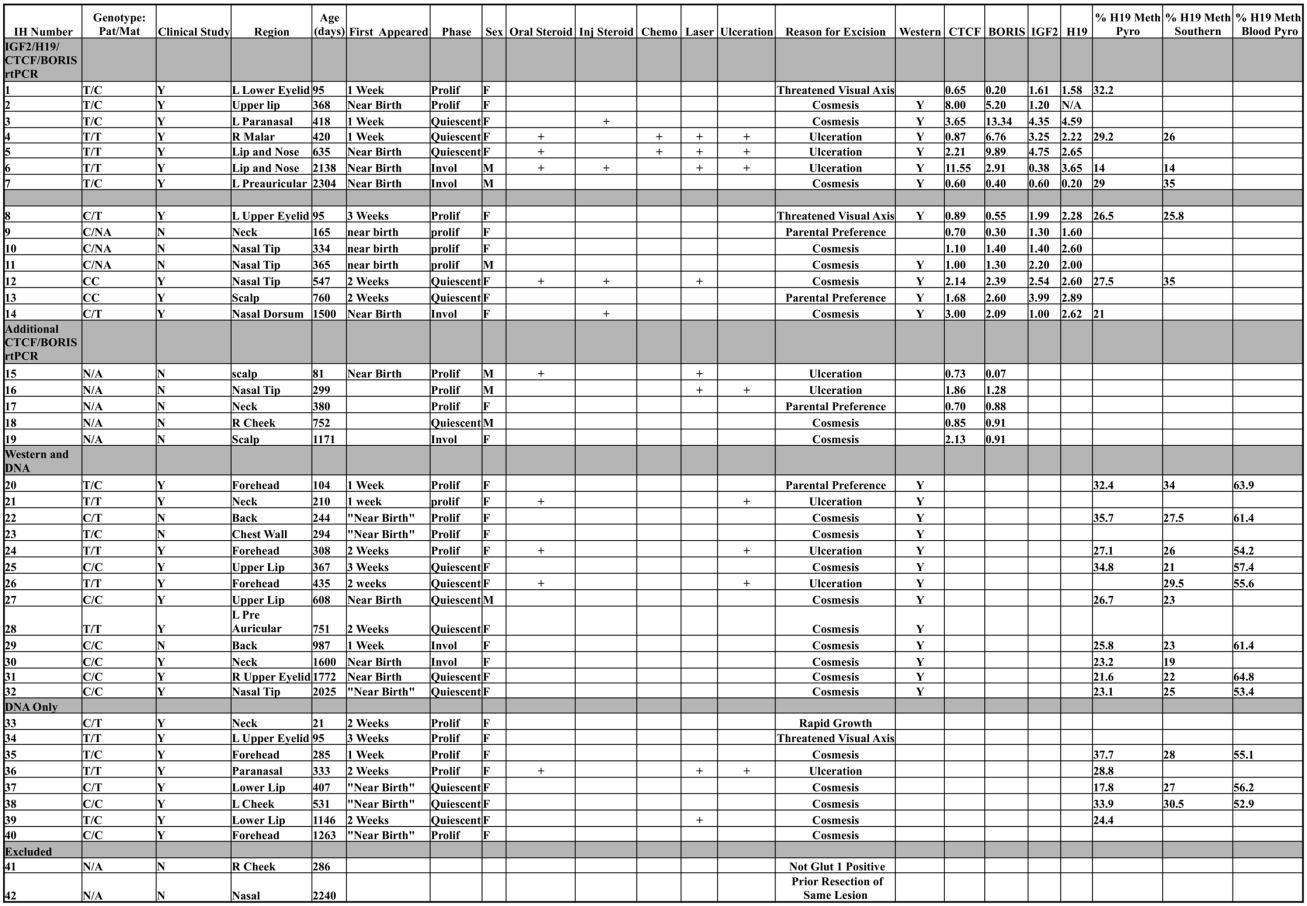
Master Data Table. All samples are assigned arbitrary numbers for ease of reference. Samples are categorized according to which set of experiments were performed, then by paternal/maternal genotype regarding the IGF2 rtPCR experiment. All sub categories are then sorted by age at resection. All quantitative data is collated with clinical descriptors. Please see methods section under specimen collection for details regarding the selection of individual samples for each experiment.

**Fig 2:**
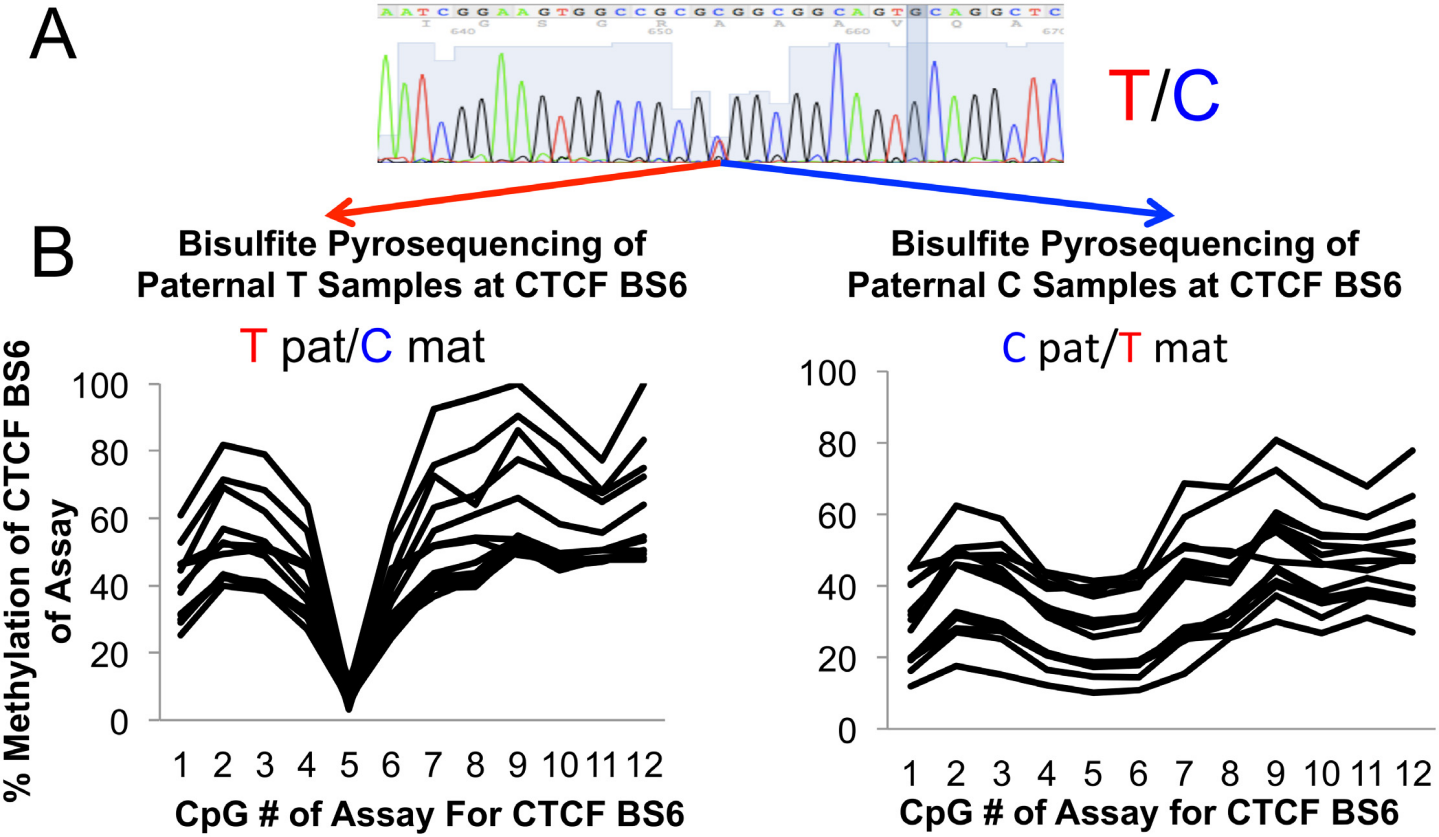
Deducing Parental Contributions From Direct Sequencing and Bisulfite Pyrosequencing. **Fig 2A**: 29 patients were genotyped via direct sequencing of blood samples for a known polymorphism within the core CTCF BS6 sequence (rs10732516.) All homozygous genotypes could be deduced from this information alone. **Fig 2B**: All samples (heterozygotes and homozygotes) were subjected to bisulfite conversion and quantitative methylation sensitive pyrosequencing. Methylation occurs only on the paternal chromosome for CTCF BS6. In normal tissue, such as patient matched control blood, this assay is capable of isolating the genotype of the paternal chromosome. As thymidine cannot be methylated, those individuals with a paternal T at rs10732516 were not methylated at CpG#5. Paternal C carrying individuals were methylated at CpG#5. Thus, the maternal and paternal contribution to CTCFBS6 can be deduced. This assay sidesteps the need for directly sequencing parents’ DNA and eliminates the potential ambiguity ensuing from hetrozygous parents. Note: The methylation values of this assay are subject to primer bias, Tost et al (25.) This is evident by the 3 distinct groupings of methylation levels, which are artifactual.

**Fig 9:**
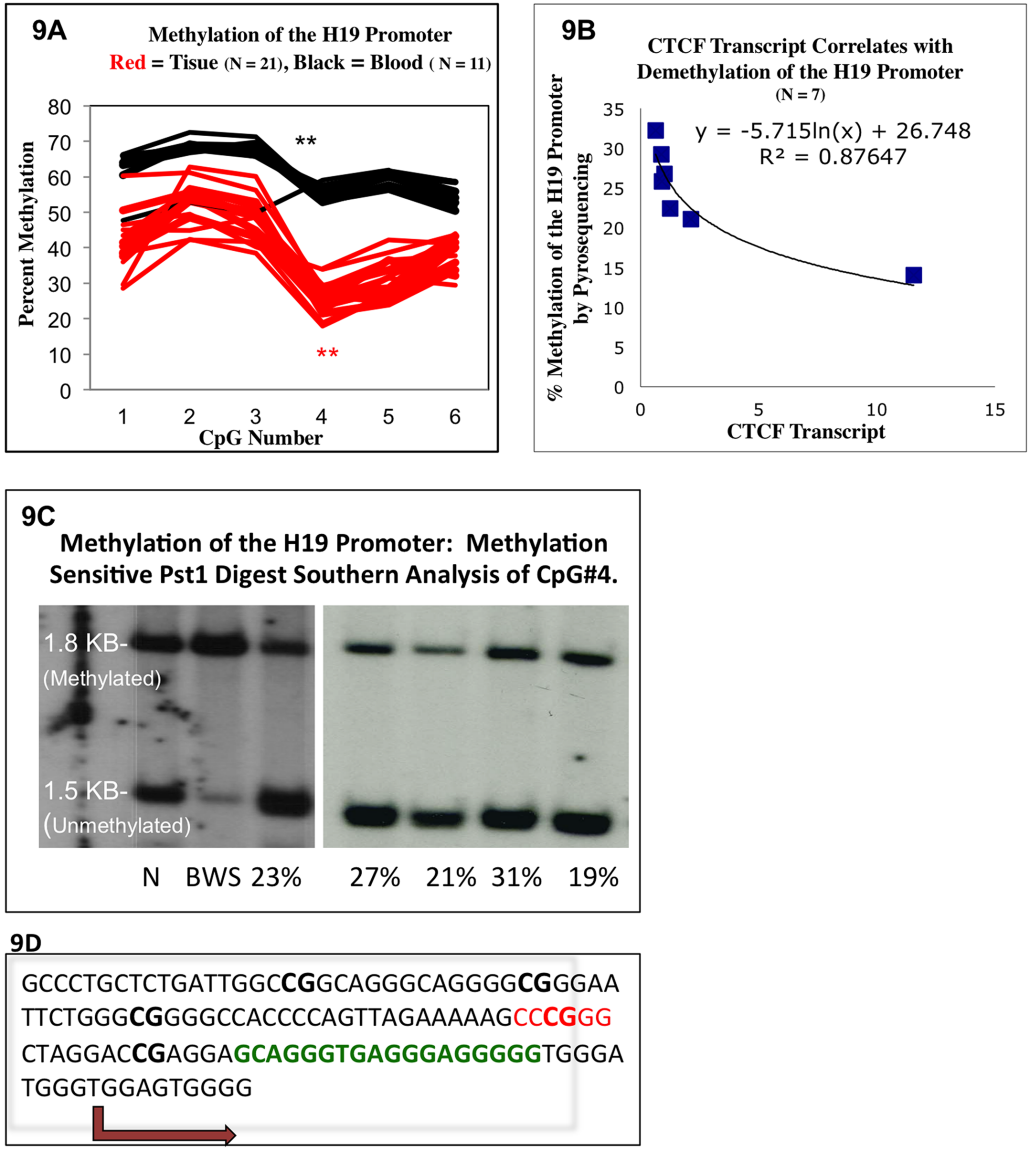
CTCF Expression and H19 Promoter Methylation. **Fig 9A**: Increased CTCF transcript level correlates with demethylation of the H19 Promoter. Those samples with the highest CTCF expression were the least methylated ranging from 34% to 14%. However, demethylation of the H19 promoter did not correlate strictly with H19 transcript expression (S6 Supplementary Information). **Fig 9B and 9C**—The H19 promoter (see **Fig 9D**) is hypomethylated, demonstrated by bisultife converted pyrosequencing (9B) and methylation sensitive restriction digest with southern hybridization (9C.) 25 IH samples, and 13 matched blood controls were subjected to bisulfite converted pyrosequencing. 13 IH samples and 13 matched blood controls were subjected to southern analysis with methylation sensitive Pst1 digestion. Two representative gels show, 5 IH samples, a Beckwith-Weidman positive control and a 50% methylated normal control. **Fig 9D**: sequence showing the H19 promoter—CpG#4 of the bisulfite sequencing test corresponds to the CCCGGG Pst1 digestion site of the Southern analysis. Other CpG’s tested are in bold. This CpG is in close proximity to the transcription start site of H19 (blue arrow) and an overlapping putative CTCF binding site identified by positional weight matrix analysis.

**Fig 11:**
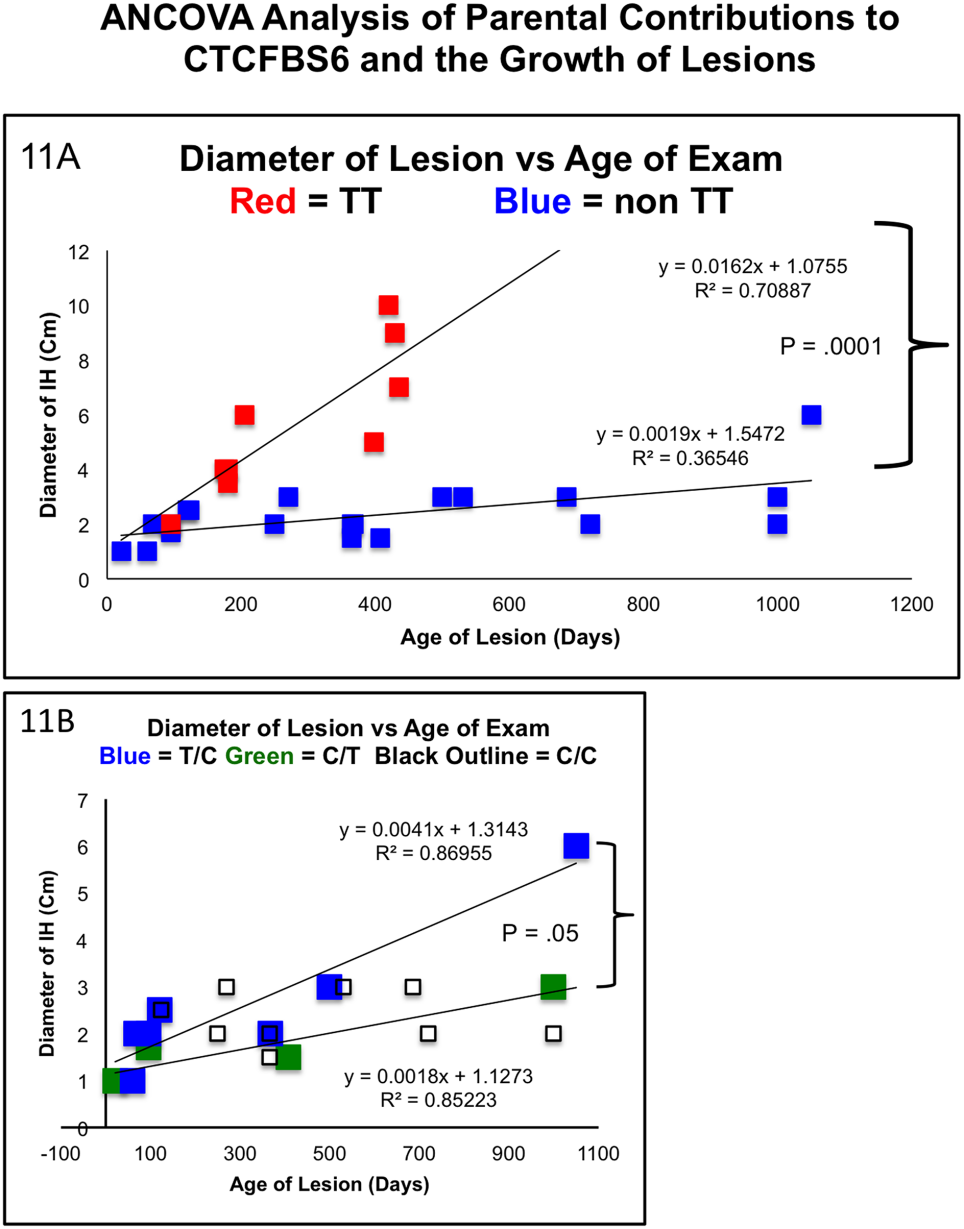
Clinical Correlation of Hemangioma Growth Rates with Parental Contributions to CTCF BS6. **Fig 11A**: This retrospective analysis of 29 samples, 9 TT, 20 non TT, demonstrates significantly distinct growth curves over a large age range. The ANCOVA model has identified age as a predictor of size p = .0007. The association between tumor size and age is significantly different among the genotypes of TT, C/T, T/C and CC p < .0001. Of Note the paternal contribution is presented first and the maternal is second. The interaction terms of parentally specific genotypes allowed us to test if the slopes of the curves between tumor size and age are different among the genotypes. This analysis indicted that an increase in 1 day of age is associated with .016cm of growth in the TT group. This is significantly higher than the non TT group p = .0019. **Fig 11B**: Growth analysis focusing on the “non T/T” group. Each non TT growth curve varied independently and significantly from the TT samples (CC vs. TT: P<0.0001, CT vs. TT: P<0.0008, TC vs. TT: P = 0.0025). Furthermore, these data suggest parent of origin specific effects as those samples with identical genotypes but opposite parental contributions displayed statistically significant differences in growth curves. The paternal T/maternal C genotype grew at approximately twice the rate as their paternal C/ maternal T carrying counterparts (p = .05). The homozygous C group appeared to have a roughly flat growth rate between the heterozygotes and did not significantly vary with either heterozygote group (CC vs. C/T p = .99, CC vs. T/C p = .74)
