# Supplementary figures and images for: A Common Polymorphism within the IGF2 Imprinting Control Region Is Associated with Parent of Origin Specific Effects in Infantile Hemangiomas
Source: PLoS One. 2015 Oct 23;10(10):e0113168. doi: 10.1371/journal.pone.0113168 (PMC4619854; doi:10.1371/journal.pone.0113168)

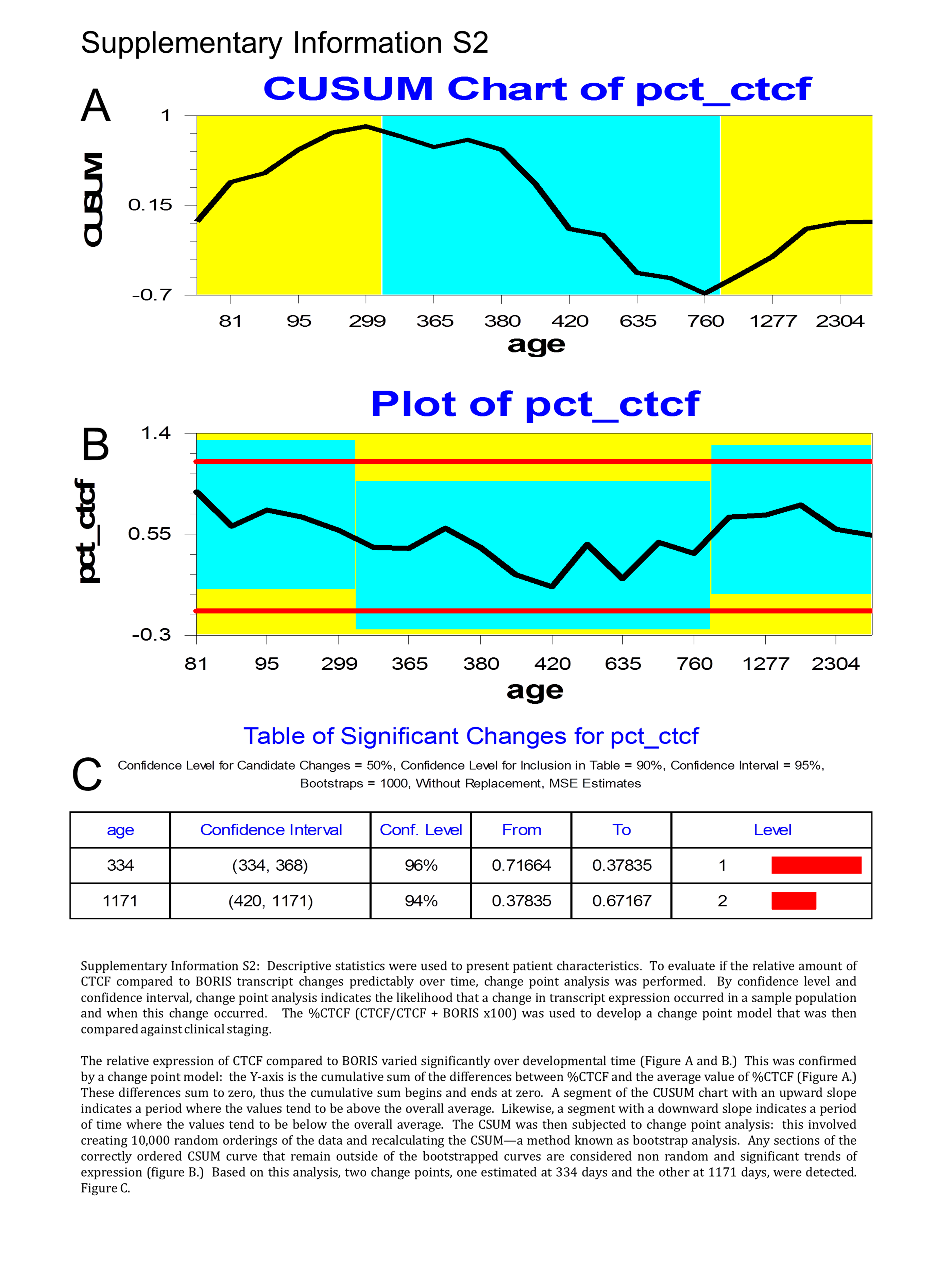

Supplement: S1 Supplementary Information — (TIF) [file pone.0113168.s001.tif]

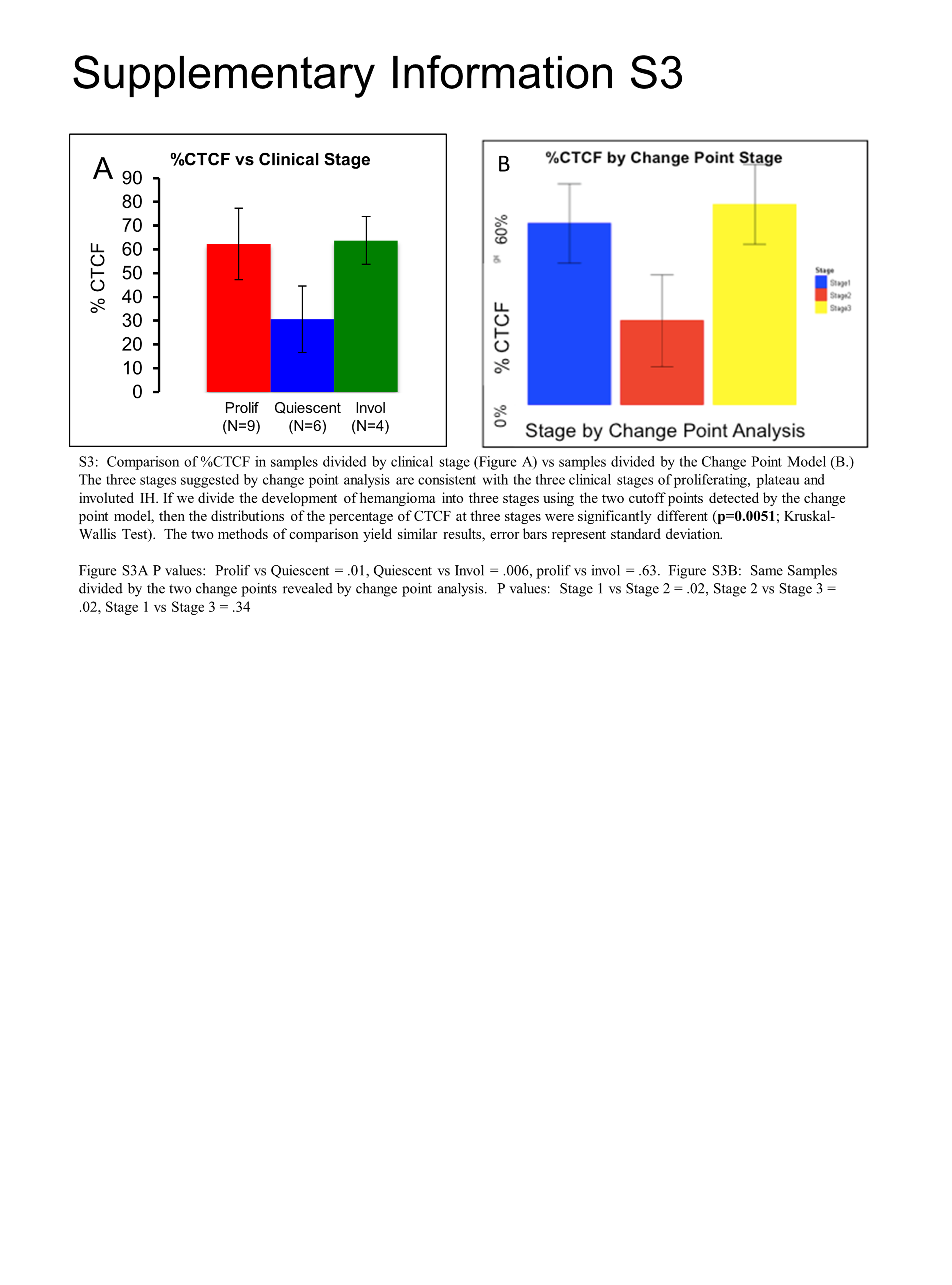

Supplement: S2 Supplementary Information — (TIF) [file pone.0113168.s002.tif]

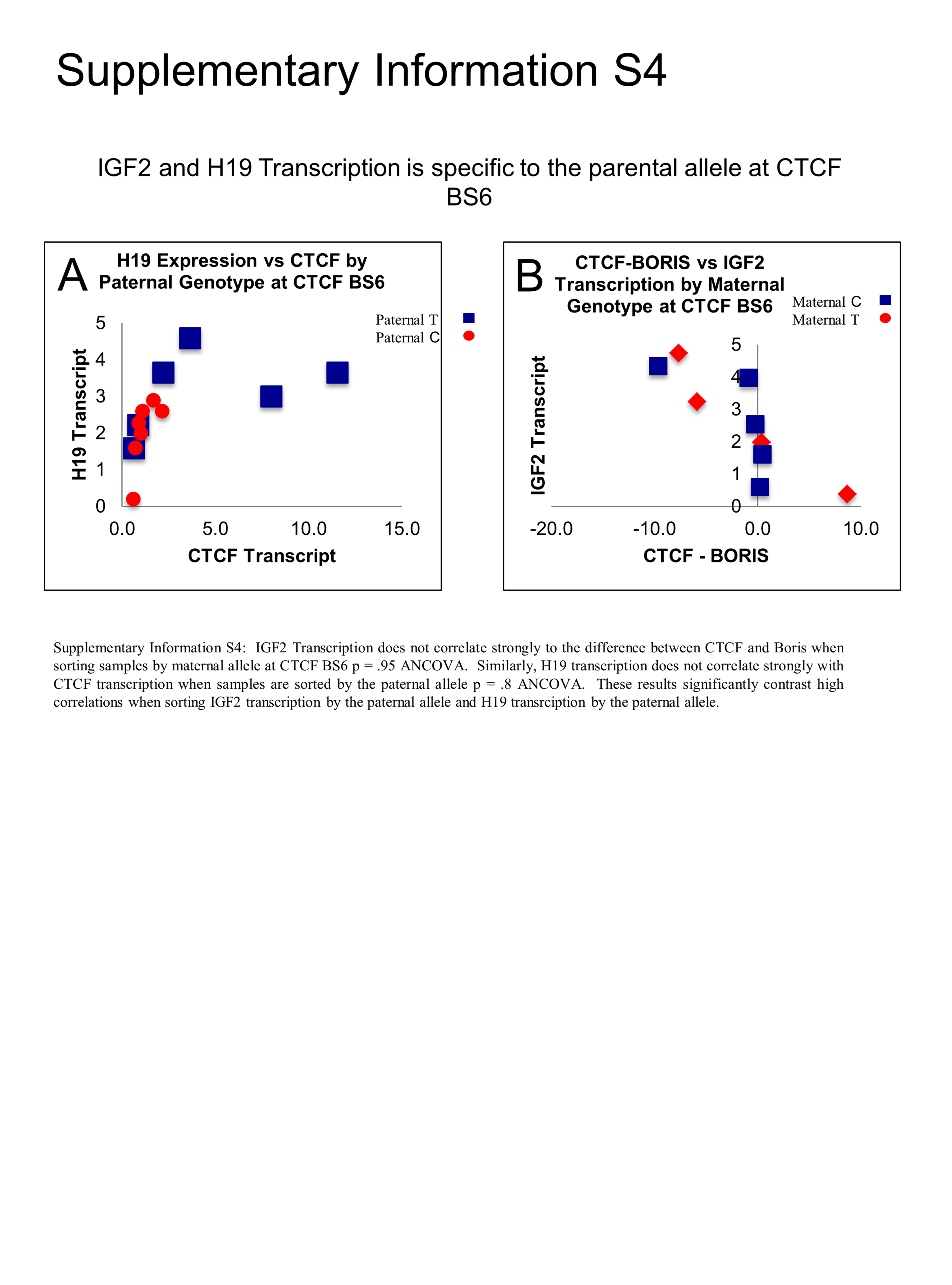

Supplement: S3 Supplementary Information — (TIF) [file pone.0113168.s003.tif]

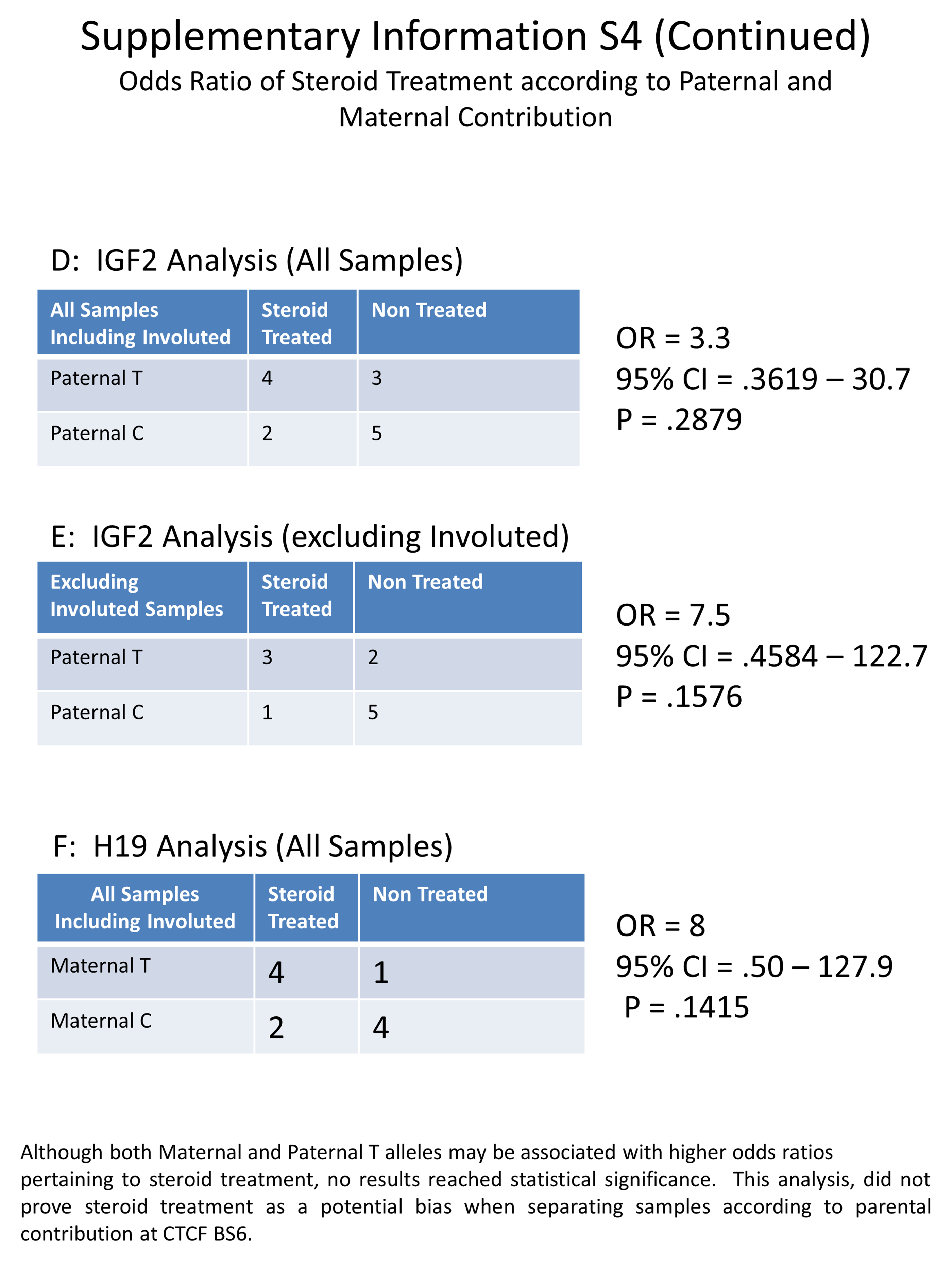

Supplement: S4 Supplementary Information — S4A—IGF2 by Maternal Genotype. S4B—H19 by Paternal Genotype. S4D-F—Odds Ratios for Potential Steroid Treatment Bias by CTCF BS6 Parental Contribution. (TIF) [file pone.0113168.s004.tif]

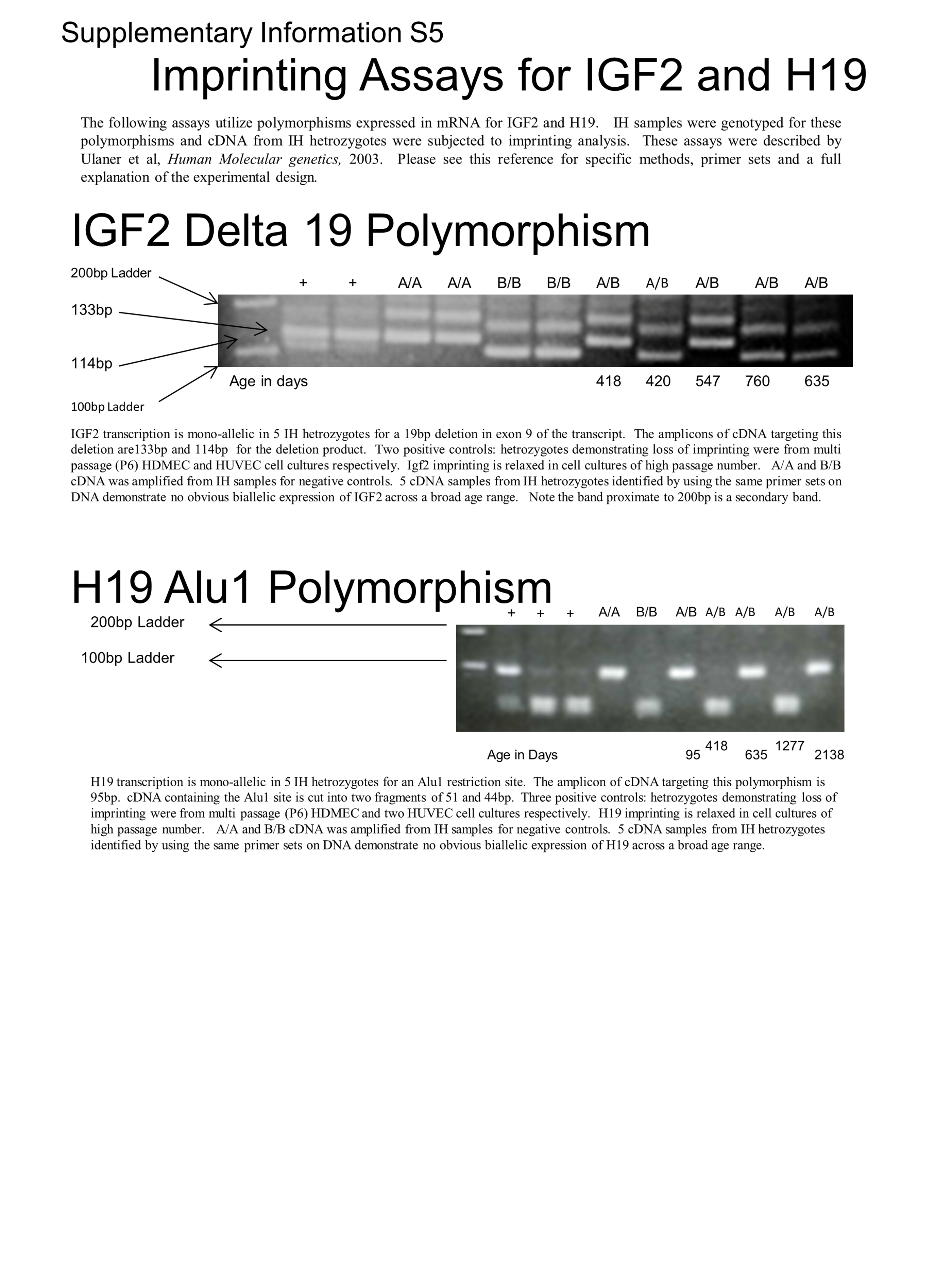

Supplement: S5 Supplementary Information — (TIF) [file pone.0113168.s005.tif]

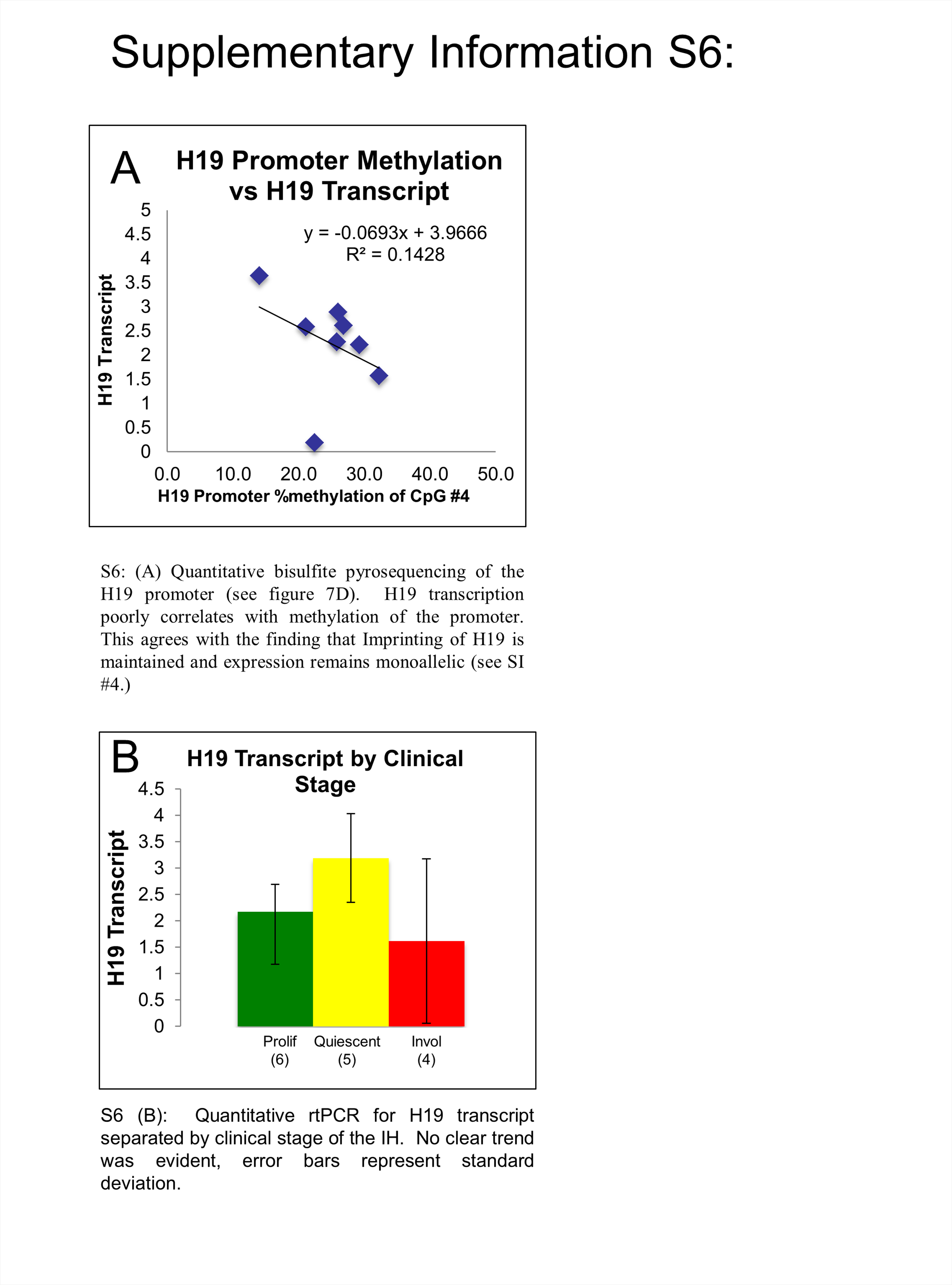

Supplement: S6 Supplementary Information — (TIF) [file pone.0113168.s006.tif]

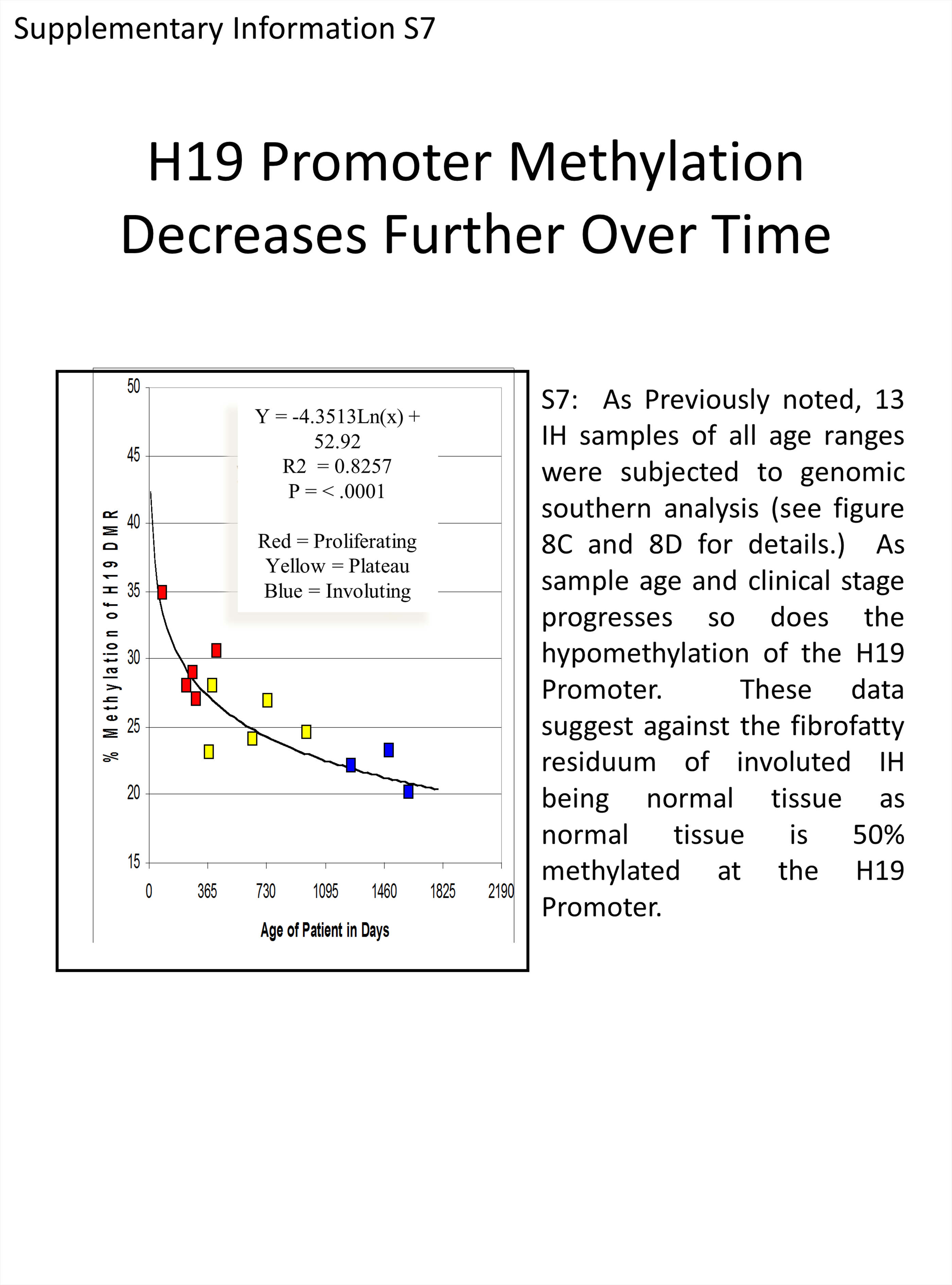

Supplement: S7 Supplementary Information — (TIF) [file pone.0113168.s007.tif]

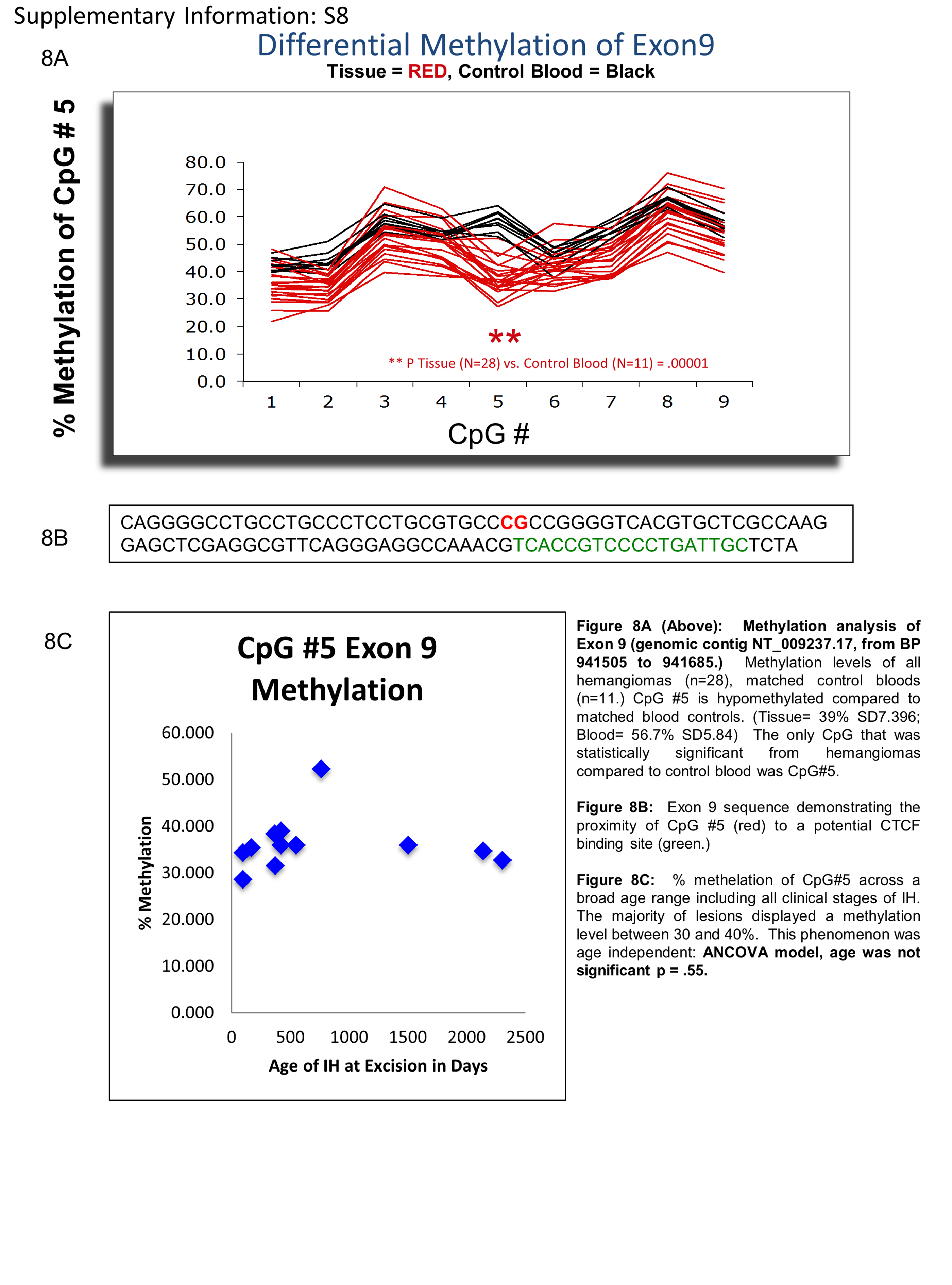

Supplement: S8 Supplementary Information — (TIF) [file pone.0113168.s008.tif]

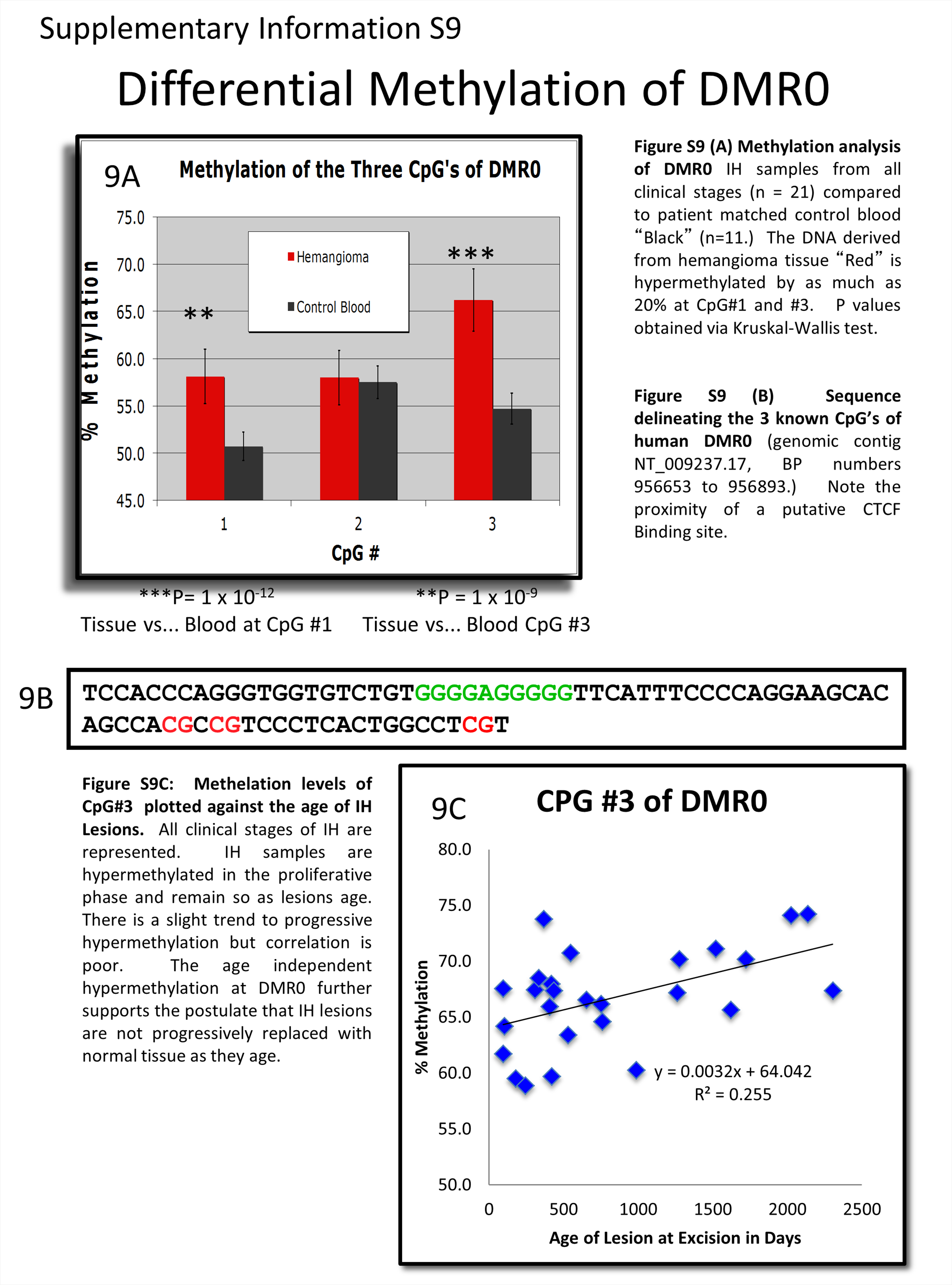

Supplement: S9 Supplementary Information — (TIF) [file pone.0113168.s009.tif]
